# Rat models for arterial calcification associated with chronic kidney disease: a systematic review and meta-analysis

**DOI:** 10.1186/s12872-025-05471-4

**Published:** 2026-01-27

**Authors:** Nicolas Hense, Melina Stein, Nikolaus Marx, Nadine Kaesler, Claudia Goettsch

**Affiliations:** 1 Department of Internal Medicine I – Cardiology, Medical Faculty, RWTH Aachen University, Pauwelsstrasse 30, Aachen, 52074 Germany; 2 Department of Internal Medicine II – Nephrology, Medical Faculty, RWTH Aachen University, Pauwelsstrasse 30, 52074 Aachen, Germany; 3 Institute of Physiology, Medical Faculty Carl Gustav Carus, Technical University Dresden, Fetscherstr. 74, Dresden, 01307 Germany

**Keywords:** Arterial calcification, Rodent model, In vivo, CKD

## Abstract

Arterial calcification represents a major burden in chronic kidney disease (CKD) and is an independent risk factor for cardiovascular diseases (CVD). Rodent models are essential for preclinical research on arterial calcification mechanisms and potential therapeutic interventions, permitting longitudinal analysis of disease progression, which is ethically unfeasible in humans. In this study, we specifically focused on rat models as representative rodent models, given their well-established use in cardiovascular research. Through systematic literature screening, we identified 470 studies employing rat models to investigate arterial calcification, with these models designed to simulate various pathological conditions. Our analysis revealed that arterial calcification was predominantly induced through kidney impairment, vitamin D overload, or mechanical and chemical vessel damage. Female rats were significantly underrepresented across studies, highlighting a considerable sex bias in experimental design. Particular emphasis was given to CKD-associated arterial calcification models, which accounted for about 60% of the identified studies. A meta-analysis of 67 CKD-related studies identified several significant factors influencing arterial calcification. Dietary phosphate concentration, male sex, and the use of Sprague-Dawley rats were associated with enhanced arterial calcification development. Nephrectomy-based approaches demonstrated superior efficacy and reliability in arterial calcification induction compared to adenine-based models. However, the analysis was limited by substantial heterogeneity across studies, potential publication bias, and inconsistent reporting of experimental parameters.

These findings underscore the critical need for standardized reporting of experimental procedures and results to enhance the applicability of animal studies in meta-analyses and improve their translational value.

**Supplementary Information:**

The online version contains supplementary material available at 10.1186/s12872-025-05471-4.

## Introduction

Arterial calcification is a complex pathophysiological disorder characterized by the progressive accumulation of mineral crystals within the arterial walls. This mineralization process begins with the deposition of amorphous calcium-phosphate complexes, which subsequently crystallize into hydroxyapatite [1]. Arterial calcification has emerged as an independent risk factor and predictor for cardiovascular disease and all-cause mortality and has recently gained attention as a parameter for risk stratification [2–4]. Calcification appears in two forms. Intimal calcification, linked to atherosclerotic lesions and caused by inflammatory processes, and medial calcification (Mönckeberg sclerosis), often seen in type 2 diabetes and chronic kidney disease (CKD) [5]. Yet, both forms can also coincide, for example, in patients with CKD-related atherosclerosis [6]. 

The link between declining kidney function and arterial calcification is robust in advanced CKD stages. Epidemiological data show that about 40% of patients with CKD stage 3 have coronary artery calcification, compared to only 13% of age-matched individuals without CKD [7]. This association arises from the complex dysregulation of mineral metabolism in CKD, involving disrupted calcium and phosphate balance and changes in key regulatory hormones, including parathyroid hormone (PTH), vitamin D (VitD), and fibroblast growth factor-23 (FGF23). These metabolic disturbances, known as CKD-mineral bone disorder (CKD-MBD), create a conducive environment for arterial calcification. The relationship seems bidirectional, as increased coronary artery calcification accelerates the progression of existing CKD [8]. Furthermore, this link is observed beyond CKD, with similar associations found in patients with normal kidney function [9]. 

The interaction between CKD and arterial calcification requires thorough research into both the underlying mechanisms and possible treatments, especially given the current lack of effective therapies for arterial calcification [10] both within and beyond the CKD context. Animal models serve as essential tools, providing a controlled environment to study the various clinical manifestations of arterial calcification. Rats and mice are commonly used as rodent models due to easy handling, low cost, and short gestation periods. For certain applications, rats are preferred over mice because their larger body size makes surgical interventions or tissue handling easier [11]. However, the heterogeneity of available models poses a challenge for standardization and comparison. This systematic review and meta-analysis aims to offer an overview of existing rat arterial calcification models and to examine methodological approaches in studies of CKD-associated arterial calcification. We performed quantitative analyses of arterial calcification severity and assessed the comparability of study designs. This structured approach will help improve understanding of available experimental tools and their uses in arterial calcification research, ultimately supporting more effective translational studies in the field.

## Materials & methods

### Protocol and registration

The study was registered with PROSPERO at https://www.crd.york.ac.uk/prospero/ (CRD42021269285). The study protocol, including inclusion criteria and methodology, was established before starting the review, and minor modifications were documented throughout the process. The study followed the updated 2020 Preferred Reporting Items for Systematic Reviews and Meta-Analyses (PRISMA) guidelines [12]. 

Clinical trial number: not applicable.

### Eligibility criteria

Studies were considered eligible for inclusion if they met three main criteria: (1) peer-reviewed articles published in English; (2) use of an in vivo arterial calcification model in mice or rats; and (3) calcification as a primary, intentional outcome of the experimental intervention, not an incidental or secondary effect of unrelated treatments.

Arterial calcification was defined as mineralization in major arteries (aorta and primary branches) and heart valves. Only direct assessments of calcification were considered valid, including histological staining, calcium elution, or tissue-nonspecific alkaline phosphatase (TNAP) activity measurements (excluding TNAP expression levels). Indirect measures, such as serum or plasma calcium or TNAP levels, were excluded from the analysis.

### Search strategy

The literature search was performed using three electronic databases: PubMed, Web of Science, and Embase. The initial search strategy included both rat and mouse studies on arterial calcification because we aimed to get a complete overview of the current experimental landscape before defining the final scope of the study. Due to the high number of eligible studies (650 mouse and 470 rat publications), we limited our analysis to rat models to ensure feasibility and to focus on evaluating model features and implementation strategies.

The search strategy employed the following combination of terms for screening titles, abstracts, and articles in press: (“cardiovascular” OR “arterial” OR “cardiac” OR “vascular” OR “valve”) AND (“calcification” OR “mineralization”) AND (“rat” OR “rats” OR “mouse” OR “mice” OR “rodent” OR “murine”). The initial search was conducted on October 15th, 2021, with an update on October 1st, 2024.

### Study selection

Two examiners independently conducted abstract and full-text screening in sequence. Any disagreements were resolved through consultation with a third reviewer for the final decision.

### Data extraction

Data extraction focused on experimental groups where calcification was actively induced. Groups that received additional therapeutic interventions were excluded from further analysis. Multiple experimental groups within individual studies, representing different variations of the arterial calcification model, were treated as separate entities (“study groups”) to reflect model differences and intervention patterns accurately.

Extracted data included general parameters (sex, strain, age, weight), study design details (model description, duration, diets, adenine, VitD, nicotine, and warfarin/vitamin K levels), bone morphology, PTH and FGF23, and arterial calcification outcomes (assessment method, tissue analyzed, time point).

For the meta-analysis, tissue calcium measurements were chosen as the primary outcome. Studies were included only if they specified sample size, included non-calcified controls, and reported standard deviation or standard error of the mean. Data from the figures were extracted using WebPlotDigitizer Version 4.5 when not available in text form. Authors were contacted regarding missing information, but only 5 of 66 requested data sets were provided. Incomplete datasets were excluded from the meta-analysis.

### Synthesis of results and statistics

Using the reported tissue calcium concentrations, the standardized mean difference (SMD) between the model and control groups was calculated with Hedges’ g, assigning study weights through inverse variance weighting. SMD values were used to represent the calcification effect size throughout this analysis.

Forest plots with 95% confidence intervals were generated using the 'metafor' R package [13]. A random effects model was employed for statistical analysis, with heterogeneity assessed using Cochran’s Q Test and I². Publication bias was assessed with Egger’s test, and meta-regression was used to analyze the relationships between independent variables and SMD.

The meta-analysis was limited to end-point aortic calcium data from studies showing significant kidney impairment (confirmed through creatinine, urea, BUN, or GFR measurements). Variation coefficients were calculated from raw calcium measurements as the standard deviation_(calcium measurement)_ / mean_(calcium measurement)_ * 100 (for model and control group). Additional visualizations were generated using GraphPad Prism 10.

### Risk of bias assessment

Risk of bias was assessed using SYRCLE’s risk of bias tool for animal studies [14]. Articles were evaluated across multiple bias domains and classified as having low, medium, high, or unclear risk of bias.

Ethics, Consent to Participate, and Consent to Publish declarations: not applicable.

## Results

### Study selection

**Fig. 1:**
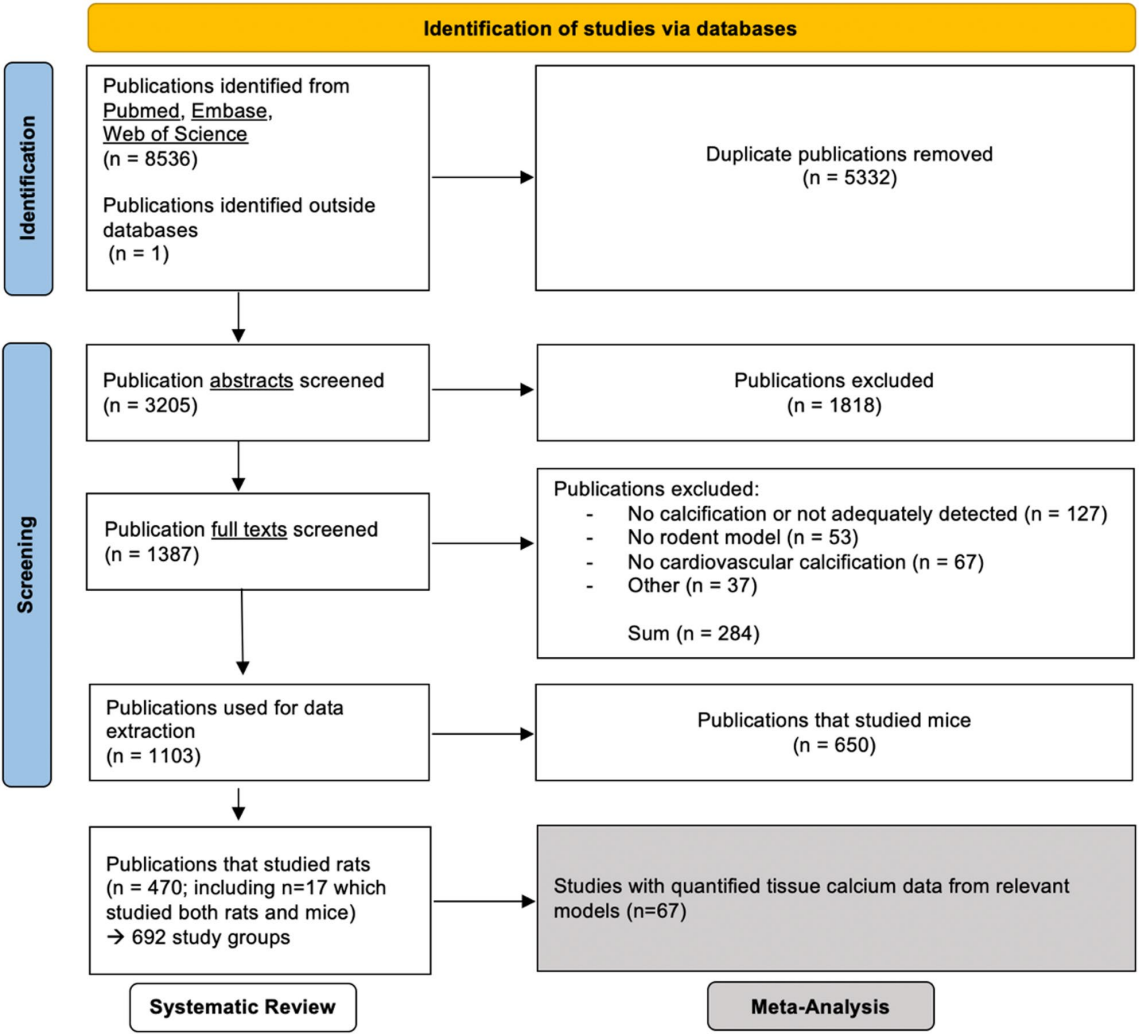
Study selection process and number of included studies for the systematic review and meta-analysis. The last literature search was performed on 01.10.2024

The study selection process adhered to PRISMA guidelines and is shown in the flow diagram **(**Fig. 1**)**. The initial screening of titles, abstracts, and full texts identified 1,103 eligible studies, including 650 using mouse models, 470 using rat models, and 17 employing both species. To ensure analysis consistency and better manage data variability, we focused our systematic review on studies that used only rat models (Supplementary Table 1).

The temporal analysis revealed a substantial increase in rat-based arterial calcification studies since 2000 **(**Supplementary Fig. 1a), with contributions from diverse geographical regions (Supplementary Fig. 1b). The risk for various biases remained unclear for most categories due to unspecified information (Supplementary Fig. 1c; Supplementary Table 2).

**Fig. 2:**
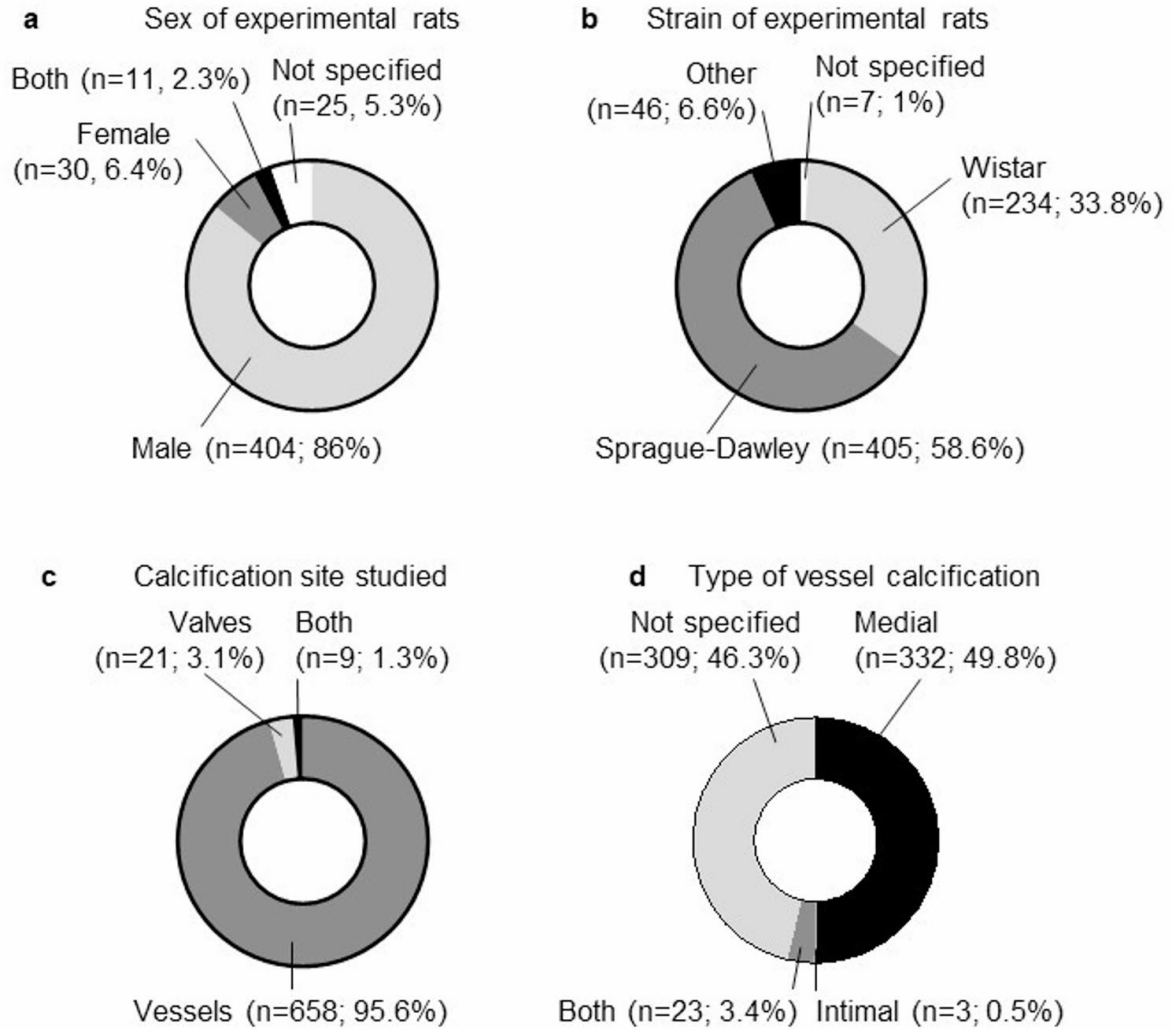
.Figure 2. General statistics of the 470 publications containing rat calcification models. a) Sex of the experimental animals overall. n indicates the number of publications. b) Strain of experimental rats. ‘Other’ includes heterozygous Han:SPRD-Cy rats (Indiana University colony)(Cy/+ IU), Dahl salt-sensitive, Dark Agouti, Fischer, Holtzmann, JCR: LA, Lewis, Simonsen albino, spontaneously diabetic Torii, spontaneously hypertensive rats, Zucker rats, and wild rat strain with Vkorc1C139/C139 mutations. n indicates the number of study groups. c) Distribution of studies investigating vessel or valve calcification. n indicates the number of study groups. Four study groups were excluded, since they did not report a calcification readout for this particular group, as opposed to parallel study groups of the experiment. d) Type of calcification described in the vessel calcification studies. The classifications refer to the categorization made by the authors of the respective studies

Analysis of the 470 rat studies revealed sex-based disparities in experimental design. Most (404 studies; 86.0%) used only male rats, while 30 studies (6.4%) used female rats, and 11 studies (2.3%) included both sexes. Sex was not specified in 25 studies (5.3%) **(**Fig. 2a**)**. Researchers commonly justified choosing male rats by citing potential confounding effects of female hormonal cycles or presumed higher susceptibility of male rats to arterial calcification [15–24]. When excluding studies that specifically examine the effects of sexual hormones, the sex distribution revealed an even greater male predominance (87.6%) (Supplementary Fig. 1d). Regarding strain selection, Sprague-Dawley (58.6% of study groups) and Wistar rats (33.8%) were the most commonly used strains, with other rat strains being used rarely **(**Fig. 2b**)**.

The anatomical focus of the studies showed that 658 study groups (95.6%) investigated vessel calcification exclusively, while 21 groups (3.1%) examined valve calcification, and nine groups (1.3%) assessed both sites **(**Fig. 2c**)**. Despite their clinical importance, models of valve calcification remain a niche within rat calcification research. As a result, later analyses primarily focused on studies of vessel calcification. Of the studies investigating vessel calcification, 49.8% of study groups demonstrated medial calcification, 0.5% intimal calcification, 3.4% showed both types, and in 46.3% of cases, the authors did not clearly specify the type of calcification **(**Fig. 2d**)**.

### Arterial calcification is induced predominantly through kidney impairment

The analysis of arterial calcification induction strategies revealed various methodological approaches across the included studies. A comprehensive summary of all identified models and their frequencies is provided in Supplementary Table 3.

Dietary manipulation of mineral content, especially phosphate and/or calcium enrichment, has become a common strategy to disrupt systemic mineral homeostasis. Our analysis revealed significant variability in the definition of ‘high’ across different studies, indicating a lack of standardized definitions. A comparison of phosphate and calcium levels revealed overlapping ranges between what is labeled as ‘high’ and control dietary levels (Supplementary Fig. 2). To establish consistent categorization standards, we set threshold values. Diets with ≥ 0.9% phosphate were classified as high-phosphate, and those with ≥ 1.2% calcium as high-calcium diets. Notably, the specific source of dietary phosphate often remained unspecified in the methodology sections.

The identified models used different primary mechanisms to induce arterial calcification, reflecting various clinical manifestations (Supplementary Table 4). These mechanisms were systematically classified based on their underlying pathophysiological principles. The most common mechanisms were associated with features of CKD-MBD, with kidney damage being the primary focus (394 study groups), followed by systemic mineral imbalance (292 study groups), and endocrine calcium-phosphate dysregulation (258 study groups)**(**Table 1**)**.

**Table 1:** Overall occurrence of each identified underlying arterial calcification induction mechanism. Total counts for each mechanism, whether occurring alone or with other factors

Arterial calcification induction mechanism	# Study groups
Kidney damage	394
Systemic mineral imbalance (diet-induced)	292
Endocrine calcium-phosphate dysregulation	258
Vessel damage (direct)	96
Hyperlipidemia	53
Inhibition of calcification antagonists	52
Diabetes	26
Estrogen reduction	13
Hypertension	12
Upregulation of calcification inducing factors	11
Age	8
Inflammation	4
Hyperuricemia (Gout)	1
Obesity	1
Overall number of study groups	662

**Fig. 3:**
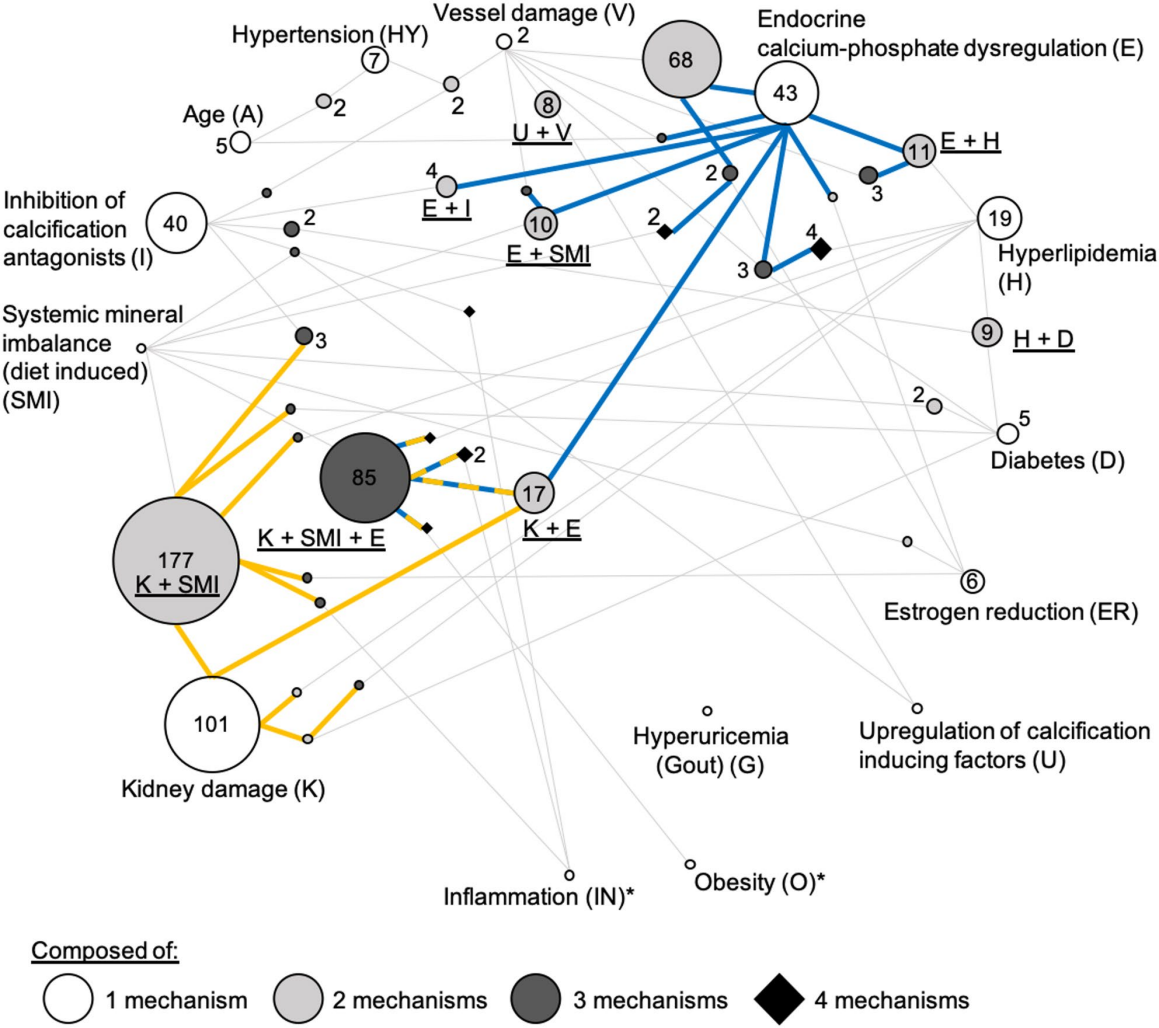
Schematic representation of the mechanistic approaches. Each circle represents a specific mechanism or a combination of several. Circle size is proportional to the number of study groups involved (indicated in or next to the circle, if not written n=1). The spatial arrangement reflects the number of mechanisms: outermost circles correspond to single mechanisms, moving inward circles represent two, three, and four mechanisms at the core, distinguished by different colors and shapes. Circles representing combinations are positioned closer to the larger “origin” mechanism(s) they originate from. Combinations are connected to their component mechanisms by gray lines, showing which mechanisms contribute to the combination. Combinations are represented by circles that are connected to their components by gray lines. Orange lines represent the network around ‘Kidney damage’. Blue lines represent the network around ‘Endocrine calcium-phosphate dysregulation’. Orange-blue dotted lines mark the overlap of both. *: Mechanisms only occur in combination and not alone – these circles therefore do not represent a study group. K = kidney damage; SMI = systemic mineral imbalance; I = inhibition of calcification antagonists; A = age; HY = hypertension; V = vessel damage; E = endocrine calcium-phosphate dysregulation; H = hyperlipidemia; D = diabetes; ER = estrogen reduction; U = upregulation of calcification inducing factors; G = hyperuricemia (gout); O = obesity; IN = inflammation

Model protocols often include multiple mechanisms to create synergistic effects, thereby increasing calcification severity. Network analysis visualization showed complex connections among these mechanisms **(**Fig. 3**)**. Kidney damage models showed variability, implemented either as standalone interventions (101 study groups) or combined with other mechanisms, especially systemic mineral imbalance (177 study groups) and endocrine calcium-phosphate dysregulation (85 study groups). Endocrine calcium-phosphate dysregulation, mainly achieved through VitD supplementation or its derivatives, was commonly used either alone (43 study groups) or with vessel damage, especially via nicotine administration (68 study groups).

The network analysis identified two main mechanistic hubs focused on kidney damage and endocrine calcium-phosphate dysregulation, around which most study groups clustered. A detailed hierarchical analysis of all mechanism combinations is provided in Supplementary Table 5. Since kidney damage-based models made up about 60% of the 662 study groups that reached this analysis stage, further analyses specifically focused on these models of arterial calcification.

### Kidney damage-associated arterial calcification is primarily modeled using adenine and subtotal nephrectomy

**Fig. 4:**
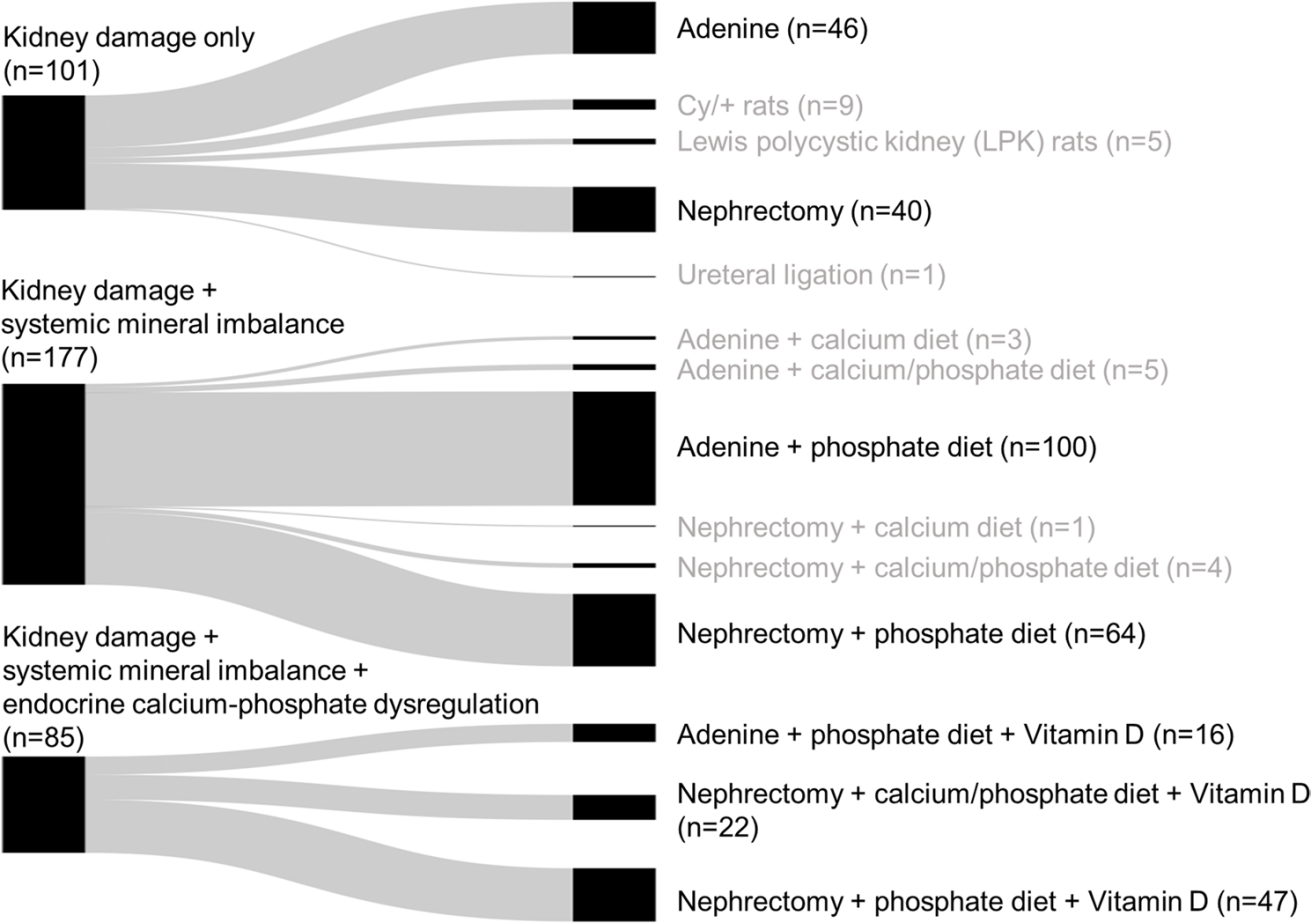
Kidney damage-based arterial calcification model approaches. Detailed listing of the exact model approaches summarized in the three biggest mechanism combination categories. n = number of study groups attributed to the respective item. Black text indicates model groups that advanced to the analysis step. Nephrectomy indicates subtotal nephrectomy approaches

Analysis of the three main mechanism combinations (kidney damage alone, kidney damage with systemic mineral imbalance, and kidney damage combined with both systemic mineral imbalance and endocrine calcium-phosphate dysregulation) identified two primary strategies for inducing kidney injury in CKD models: adenine administration and subtotal nephrectomy **(**Fig. 4**)**. Adenine was administered either through specialized dietary inclusion or direct injection, while subtotal nephrectomy involved surgically removing one kidney, along with partial resection of the opposite kidney. Alternative methods included genetic models of autosomal-recessive polycystic kidney disease, specifically Lewis polycystic kidney rats and Cy/+ rats, as well as ureteral ligation techniques.

Adenine-based and subtotal nephrectomy models showed significant methodological differences, especially in how they combined with calcium and/or phosphate-enriched diets and VitD supplementation.

To enable a detailed analysis of procedural parameters and outcomes, we chose specific model variations for thorough investigation **(**Fig. 4**)**.

### Kidney damage-associated arterial calcification models show substantial methodological heterogeneity

**Fig. 5:**
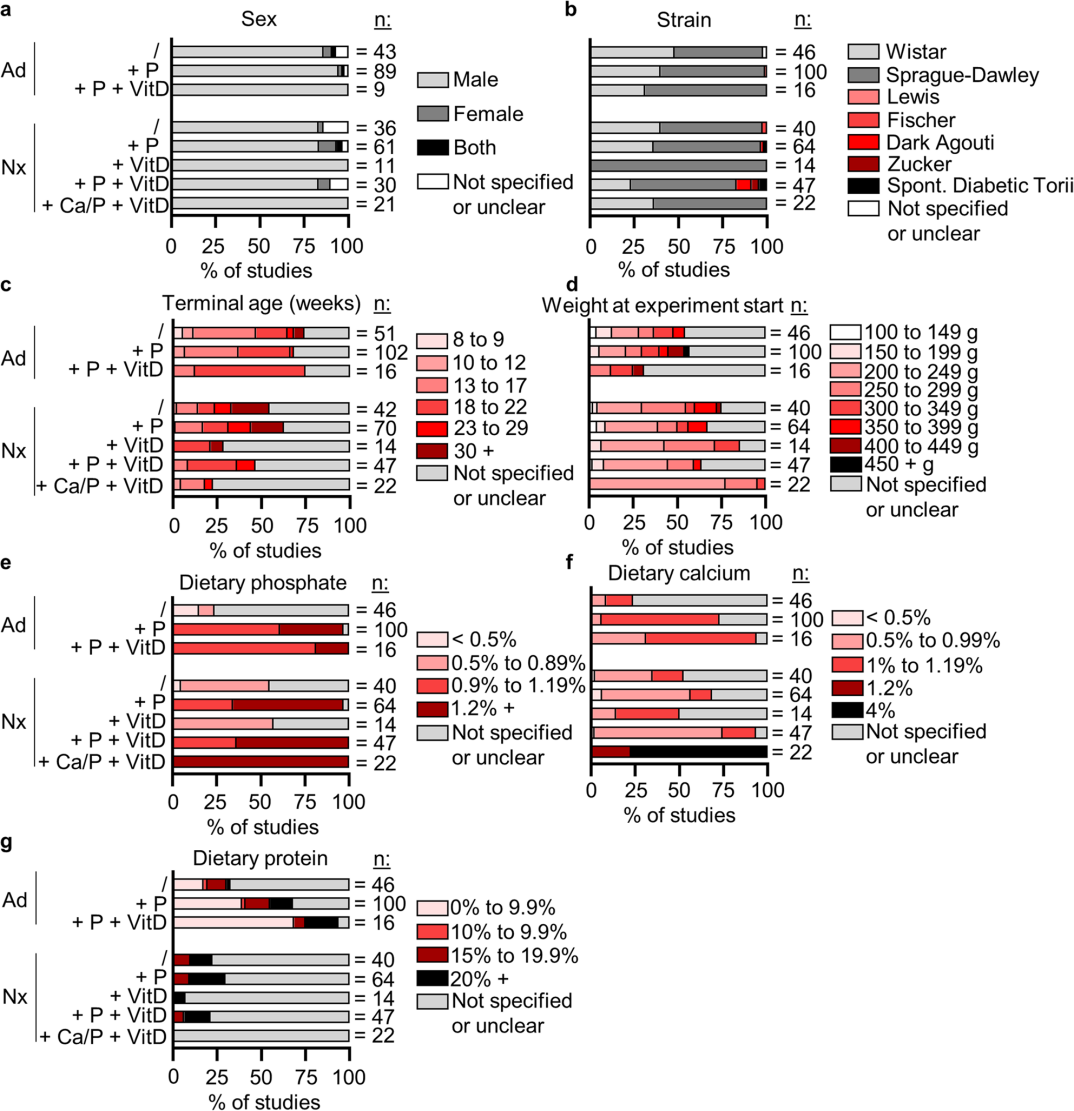
General parameters of models of arterial calcification in chronic kidney disease. **a** Sex of experimental rats. **b** Rat strain. Continuous data were categorized into ranges. If studies had more than one endpoint, several were counted. **c** Age of rats at sacrifice. **d** Mean weight of rat groups at the beginning of the study in g. **e**-**g** Dietary concentrations of phosphate, calcium, and protein were specified (in some cases, information was obtained from the manufacturer’s sheet if the exact diet was specified). n indicates the number of study groups, except in a) where the number of publications is indicated. Ad: Adenine; Nx: subtotal nephrectomy. /: no other treatment; P: high phosphate; VitD: Vitamin D (including several chemical derivatives); Ca: high calcium

Analysis of experimental parameters across the eight selected models revealed substantial methodological heterogeneity. We found a predominance of male rats across all eight models **(**Fig. 5a**)**. Strain selection showed a preference for Sprague-Dawley rats, followed by Wistar rats, with other strains used less frequently **(**Fig. 5b**)**.

There was substantial variability in both the age at sacrifice and the initial body weight at the start of the experiment **(**Figs. 5c-d**)**.

Analysis of dietary composition revealed several notable patterns. Dietary phosphate concentrations were inconsistent across models **(**Fig. 5e**)**. However, 43% to 76% of studies not explicitly based on a high-phosphate diet failed to specify dietary phosphate concentrations. Calcium supplementation patterns varied across models as well **(**Fig. 5f**)**. Protein concentration specifications were frequently omitted across studies **(**Fig. 5g**)**. Methodological variation in adenine administration, nephrectomy procedures, and VitD supplementation is provided in the supplemental material (Supplementary Fig. 3).

Given the observed variability in experimental parameters, we conducted correlation analyses to identify potential relationships between quantitative variables within study designs. Analysis of combined adenine models revealed an inverse correlation between the duration of adenine diet administration and adenine concentration (Supplementary Fig. 4a). However, adenine concentration was not associated with dietary phosphate or calcium levels (Supplementary Fig. 4b, c). In the nephrectomy models, the time from nephrectomy completion to sacrifice demonstrated inverse correlations with both dietary phosphate and calcium levels (Supplementary Fig. 4d-f). Notably, these correlations became non-significant after excluding the subtotal nephrectomy + Ca/P + VitD groups. Further analyses of individual models found no additional correlations (Supplementary Figs. 5 and 6).

The comprehensive analysis of experimental parameters across these studies shows considerable methodological diversity and a clear lack of standardized protocols, emphasizing the need for more unified experimental methods.

### A meta-analysis of arterial calcification studies revealed considerable variations in the degree of calcification

**Fig. 6:**
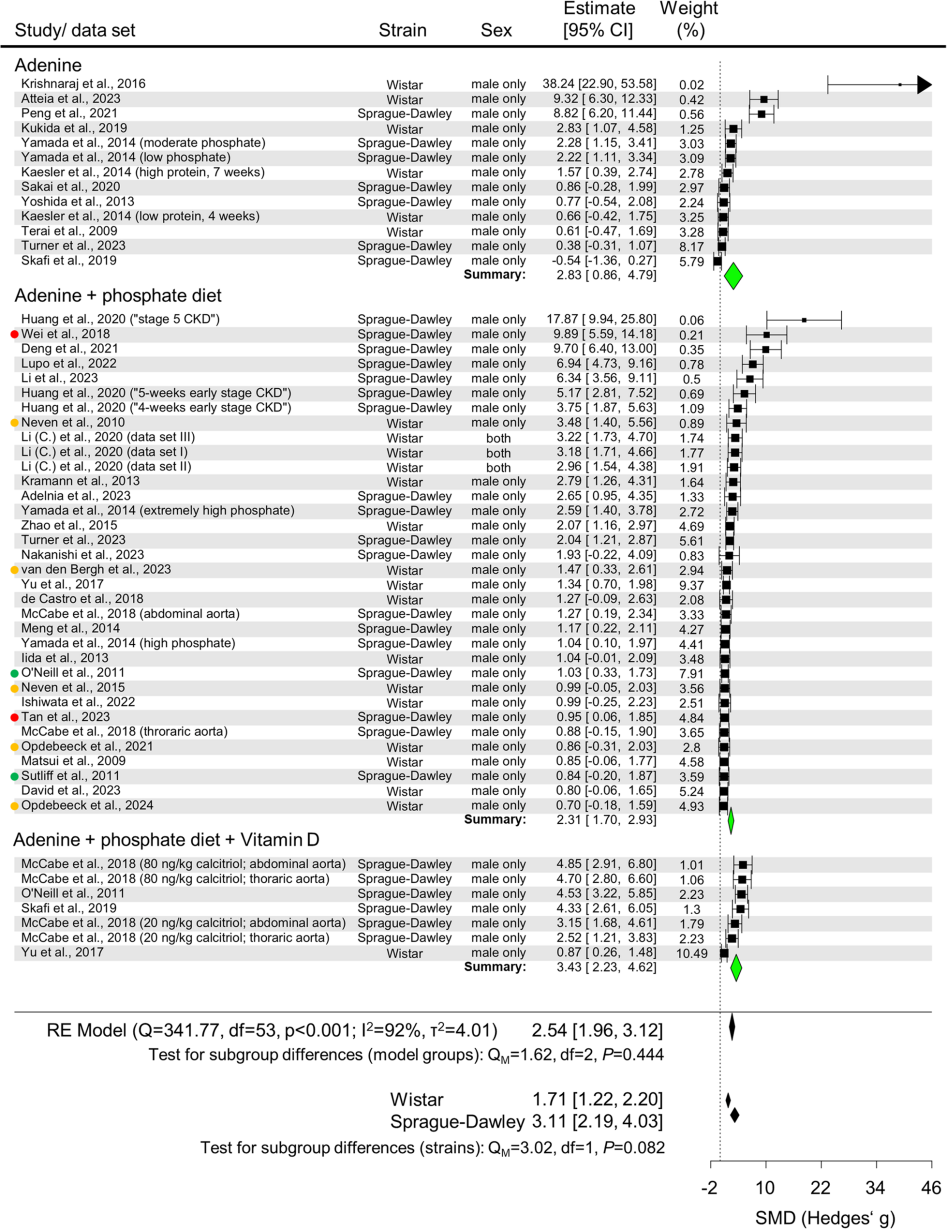
Meta-analysisof adenine models. Forrest plot of all data sets eligible for inclusion. Aortic calcium measurement data were calculated into standardized mean difference (SMD) (Hedges’g) as a metric for the effect size. SMD and the 95% confidence interval (CI) are plotted. Subgroups are defined by model type, and a test for subgroup differences was performed. A similar test was also performed for subgroups based on strain. A random effects (RE) model was chosen. Colored dots indicate if studies presumptively stem from the same institution/researchers. df = degrees of freedom

**Fig. 7:**
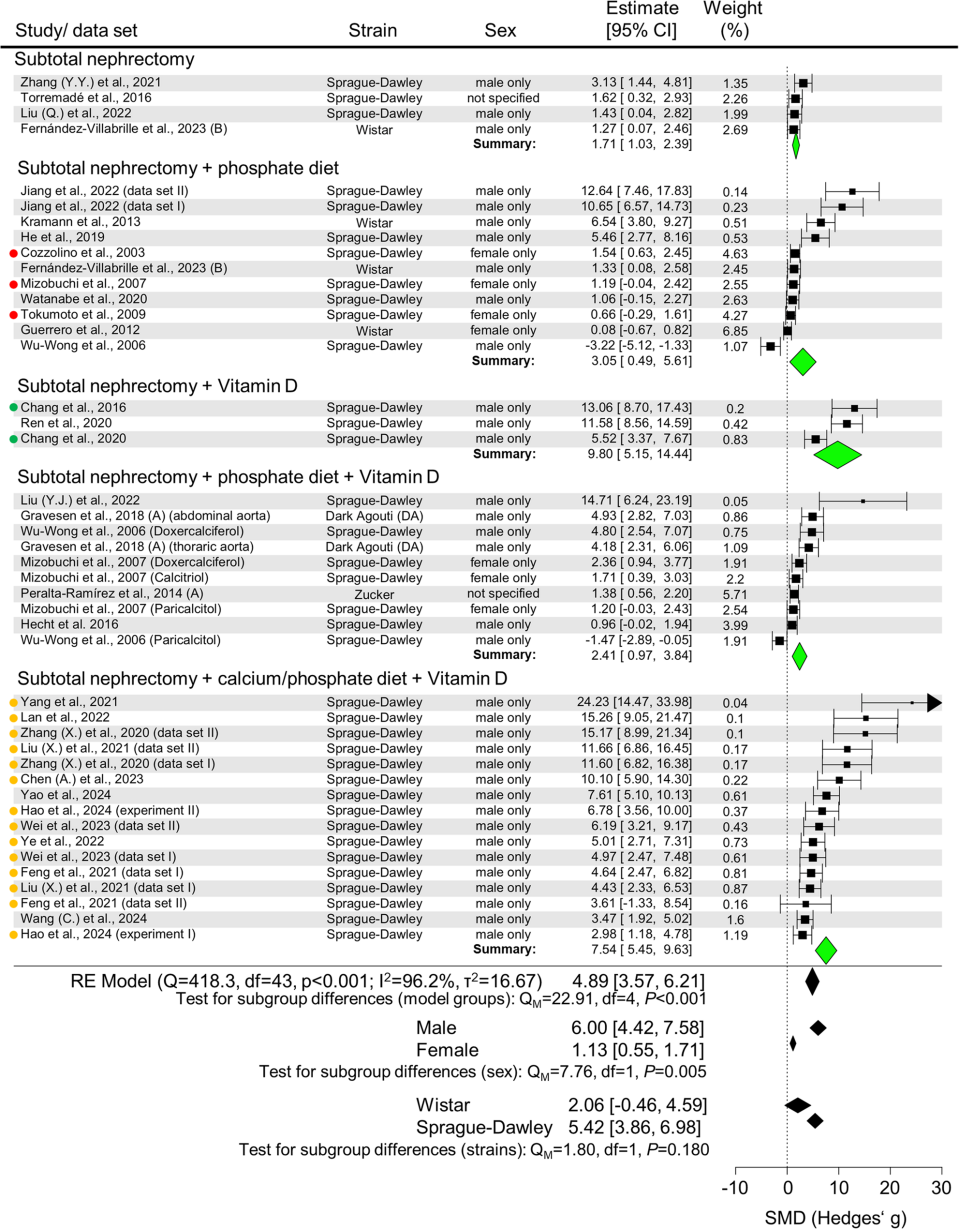
Meta-analysis of subtotal nephrectomy models. Forrest plot of all data sets eligible for inclusion. Aortic calcium measurement data were calculated into standardized mean difference (SMD) (Hedges’g) as a metric for the effect size. SMD and the 95% confidence interval (CI) are plotted. Subgroups are defined by model type, and a test for subgroup differences was performed. A similar test was also performed for subgroups based on strain or sex. A random effects (RE) model was chosen. Colored dots indicate if studies presumptively stem from the same institution/researchers. df = degrees of freedom

A meta-analysis was performed to compare the efficiency and reliability of arterial calcification induction across study groups meeting the analysis criteria **(**Figs. 6 and 7, Supplementary Fig. 7).

Analysis of adenine (Ad)-based models showed substantial variability in the effect size (SMD) of arterial calcification extent across all three subgroups **(**Fig. 6**)**. The effect estimates were similar across subgroups (SMD [95% CI] Ad: 2.83 [0.86, 4.79]; Ad + P: 2.31 [1.70, 2.93]; Ad + P + VitD: 3.43 [2.23, 4.62]), with no significant differences between subgroups (Q_M_=1.62, *P* = 0.444). Strain comparison showed higher arterial calcification in Sprague-Dawley rats compared to Wistar rats, although the difference was not statistically significant (Q_M_=3.02, *P* = 0.082) **(**Fig. 6**)**. Meta-regression analysis of potentially influential factors showed a positive association between dietary phosphate levels and the degree of arterial calcification (β = 2.781, *P* = 0.005) (Supplementary Fig. 8). The combined model analysis showed no significant effect of adenine metrics, dietary calcium, and VitD supplementation on the SMD. Individual meta-regression analyses of adenine and phosphate-enriched diets or adenine and phosphate/VitD-enriched diets supported the effect of phosphate concentration (Supplementary Fig. 9).

Nephrectomy studies showed differences in the extent of arterial calcification **(**Fig. 7**)**. While subtotal nephrectomy (SMD [95% CI] 1.71 [1.03, 2.39]), subtotal nephrectomy + P (3.05 [0.49, 5.61]), and subtotal nephrectomy + P + VitD (2.41 [0.97, 3.84]) had similar SMD estimates, significantly greater arterial calcification effects were seen in the subtotal nephrectomy + VitD (9.80 [5.15, 14.44]) and subtotal nephrectomy + Ca/P + VitD (7.54 [5.45, 9.63]) groups (qm = 22.91, *P* < 0.001). The subtotal nephrectomy + VitD subgroup included only three studies, two of which shared the same first author, and most of the subtotal nephrectomy + Ca/P + VitD studies came from a single institute. Sex-specific analysis of nephrectomy studies revealed significantly higher arterial calcification SMDs in male rats (SMD [95% CI] 6.00 [4.42, 7.58]) compared to female rats (1.13 [0.55, 1.71]) (Q_M_=7.76, *P* = 0.005), although female rats represented only 7 of 44 data sets. Sprague-Dawley rats (5.42 [3.86, 6.98]) showed a similar extent of arterial calcification compared to Wistar rats (2.06 [-0.46, 4.59]) (Q_M_=1.80, *P* = 0.180).

Meta-regression showed a positive association between dietary phosphate (ranging from 0.9 to 2.0%) and the calcification effect size (β = 5.444, *P* < 0.001) across the subgroups (excluding subtotal nephrectomy + Ca/P + VitD: β = 3.849, *P* = 0.017) (Supplementary Fig. 10). Individual subgroup analyses supported the phosphate effect only in the subtotal nephrectomy + P group (β = 6.411, *P* = 0.027) (Supplementary Fig. 11).

Analysis of all models confirmed sex-specific differences in effect size between male (SMD [95% CI] 3.91 [3.13, 4.68]) and female (1.13 [0.55, 1.71]) subgroups (Q_M_=4.04, *P* = 0.044) and strain differences between Wistar (1.75 [1.24, 2.27]) and Sprague-Dawley rats (4.29 [3.36, 5.22]) (Q_M_=7.12, *P* = 0.008) **(**Table 2**)**. Nephrectomy models demonstrated significantly higher overall arterial calcification (SMD [95% CI] 4.88 [3.57, 6.21]) compared to adenine models (2.54 [1.96, 3.12]) (Q_M_=7.06, *P* = 0.008). However, direct comparisons of matched subgroups (Ad / vs. subtotal nephrectomy /, Ad + P vs. subtotal nephrectomy + P, and Ad + P + VitD vs. subtotal nephrectomy + P + VitD) showed no significant differences, indicating that the higher SMD in nephrectomy models was mainly caused by the subtotal nephrectomy + VitD and subtotal nephrectomy + Ca/P + VitD groups. All study groups showed variability in experimental conditions, with detailed descriptions of model protocols and their corresponding control groups included in Supplementary Tables 6 and 7.

**Table 2:** Subgroup comparisons: adenine and nephrectomy models, strains, and sex

	Male SMD [95% CI)	Female SMD [95% CI)	qm	*P*-value
All model types	3.91 [3.13, 4.68]	1.13 [0.55, 1.71]	4.04	0.044
	Wistar	Sprague-Dawley		
All model types	1.75 [1.24, 2.27]	4.29 [ 3.36, 5.22]	7.12	0.008
	Adenine	Subtotal nephrectomy		
All model types	2.54 [1.96, 3.12]	4.88 [3.57, 6.21]	7.06	0.008
/	2.83 [0.86, 4.79]	1.71 [1.03, 2.39]	0.22	0.642
+P	2.31 [1.70, 2.93]	3.05 [0.49, 5.61]	0.01	0.929
+P + VitD	3.43 [2.23, 4.62]	2.41 [0.97, 3.84]	1.47	0.225

Estimates are specified as standardized mean difference (SMD) (Hedges’g) as a metric for the effect size. All comparisons: degrees of freedom (df) = 1

/: no other treatment; P: high phosphate; VitD: Vitamin D (including chemical derivatives)

Furthermore, negative SMDs, indicating less calcification compared to the corresponding control groups, were observed only in study groups serving as intermediate controls within studies that included an additional calcification-inducing treatment (e.g., Ad vs. Ad + P + VitD). We extracted data from these intermediate groups to compare them with studies that focused on a similar treatment regimen. This finding suggests that comparable negative outcomes might also occur in studies using these models as standalone interventions, but are often unreported, indicating possible publication bias. This interpretation is further supported by funnel plots and an Egger’s test, which show a general asymmetry in the data distribution (Supplementary Fig. 12).

Since arterial calcification and bone remodeling are interlinked, especially in CKD-MBD, we evaluated the reporting of non-vascular, bone-related metrics in CKD-related arterial calcification models. The minority of the studies assessed bone morphology and FGF23, while PTH was reported in some studies (Supplementary Fig. 13).

### Lower intra-variability in calcification extent in nephrectomy-based models compared to adenine models

**Fig. 8:**
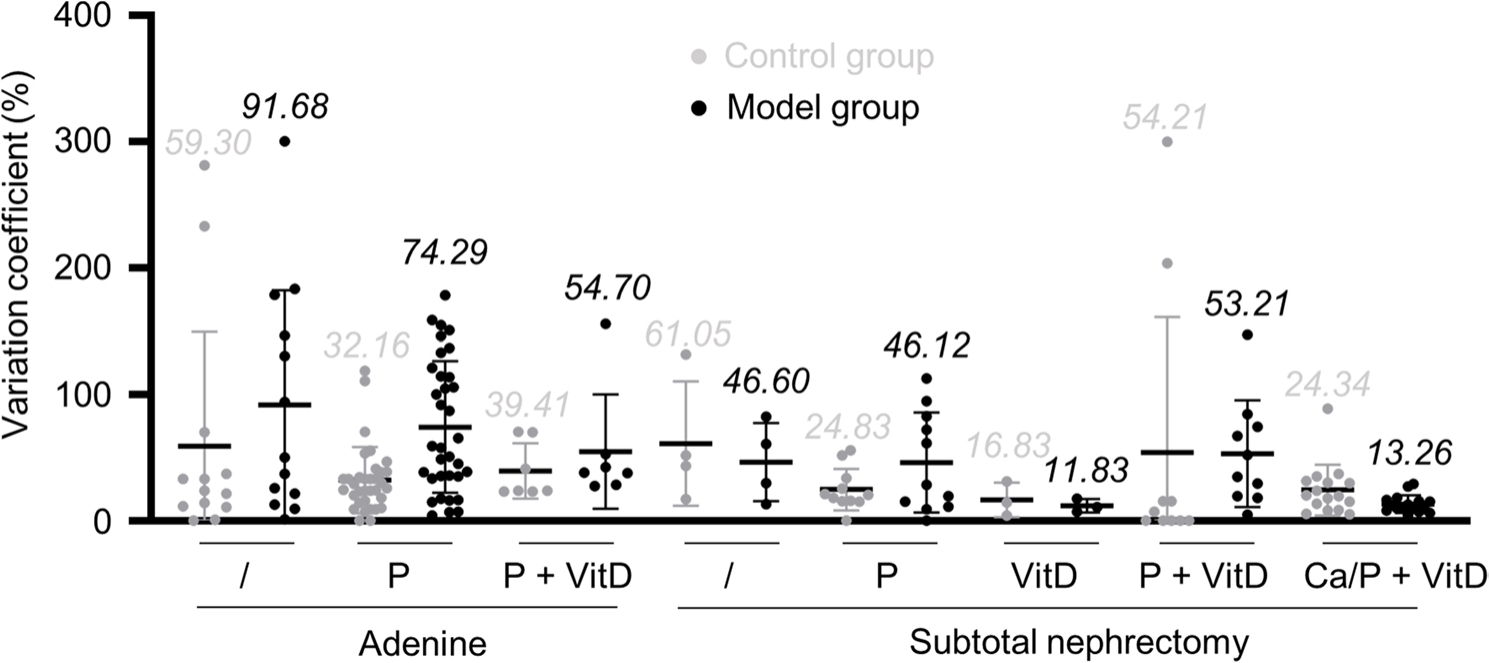
Variation in arterial calcification assessed by vessel calcium. Each data point represents the variation coefficient (calculated as standard deviation_(calcium measurement)_ / mean_(calcium measurement)_ * 100) of one data set from the meta-analysis (black) and its respective control group (gray). Mean ± SD (mean value of each group written above graph). /: no other treatment; P: high phosphate; VitD: Vitamin D (including chemical derivatives); Ca: high calcium

Finally, we assessed the variability of tissue calcium measurements within each data set to determine the consistency of different models. As a result, we calculated variation coefficients to standardize the standard deviations across all meta-analysis data sets (Fig. 8).

The analysis revealed substantial differences in the variability among the models. The adenine-only model demonstrated the highest mean variation coefficient (91.7%) and significant standard deviation across data points, indicating considerable experimental variability. In contrast, nephrectomy models demonstrated lower variation coefficients. Of note, the studies of the models with the lowest variation coefficients (subtotal nephrectomy + VitD; subtotal nephrectomy + Ca/P + VitD) of 11.8% and 13.3% were potentially performed in the same center. These findings show that nephrectomy-based models have less intra-variability of aortic calcium than adenine-based approaches.

## Discussion

This systematic review and meta-analysis offers a comprehensive overview of experimental methods used to model arterial calcification in rats. Our analysis identified various mechanisms that cause arterial calcification, which are linked to major clinical comorbidities and risk factors, including CKD, diabetes, and hypertension. Given the dominance of kidney impairment-based models, we thoroughly examined the most commonly used CKD models, revealing significant variability in experimental variables and a lack of standardized procedures across different designs. Our meta-analysis identified male sex, high dietary phosphate intake, and the use of Sprague-Dawley rats as key factors driving arterial calcification. Nephrectomy-based models tended to produce more extensive and consistent calcification compared to adenine-based models.

We identified a significant sex bias in experimental design, with female rats accounting for only 7% of the analyzed publications. Despite increased awareness of sex-specific effects in cardiovascular and kidney diseases [25–27], and a growing focus on considering sex in preclinical studies, recent publications (2020 onward) still show this disparity, with female rats making up just 5%. Our meta-analysis partially supports the commonly cited justification that male rats exhibit a higher extent of arterial calcification [20, 23]. However, this finding does not justify the systematic exclusion of female animals, especially since arterial calcification is a major cardiovascular risk factor for both sexes [28, 29]. Current evidence indicates sex-specific differences in atherosclerotic plaque patterns and development. Women exhibit more non-calcified, lipid-rich plaques than men [30]. Whether medial calcification explains sex-specific patterns remains unclear. Additionally, women with aortic stenosis tend to have less valvular calcification but more fibrosis than men [31]. Moreover, compared to women, men show a faster decline in kidney function and progress to kidney failure more frequently [32], and have a 2.2-fold higher risk of needing dialysis after hospitalization [33]. Given the known sex differences in the appearance of cardiovascular calcification in humans, studying sex-specific differences in rat arterial calcification models and their underlying mechanisms deserves greater focus to move the field forward.

Age representation in the analyzed studies presents another limitation in current modeling approaches. While over 80% of chronic kidney insufficiency patients are aged 46 years or older [27], the experimental animals in these studies were predominantly 13–29 weeks old at sacrifice, relatively young given the 2.5-year potential lifespan of Sprague-Dawley and Wistar rats. Although practical considerations and the need for accelerated disease progression influence these choices, the absence of age-related comorbidities may limit the clinical relevance of these models.

The timing and administration of high phosphate diets in these models raise questions about their clinical relevance. In CKD patients, elevated phosphate levels are usually observed in advanced stages (glomerular filtration rate < 40 ml/min/1.73 m²) [34]. While nephrectomy models simulate advanced kidney damage, making concurrent phosphate elevation physiologically plausible, the same rationale does not apply to adenine models, where kidney damage develops gradually. This gradual progression more closely resembles human pathology; however, in most cases, the high-phosphate diet is given alongside adenine. Consequently, the phosphate environment in these models does not fully reflect the clinical condition. These considerations reveal opportunities to better align animal models with human disease.

Our meta-regression analysis supports clinical observations that high phosphate levels are linked to higher mortality and worse cardiovascular outcomes in both CKD and non-CKD populations [35]. However, direct comparisons between models with and without phosphate supplementation did not consistently show increased calcification, though small sample sizes in some subgroups suggest careful interpretation. The impact of dietary phosphate source and protein composition on phosphate bioavailability [36–38], highlighted in recent studies, could not be systematically evaluated due to insufficient reporting of nutritional details. This limitation applies to all dietary components, and many studies provide only generic diet descriptions. Lee et al. (2023) recently reviewed the significant differences in the composition of commonly used chow diets and their impact on the animals’ metabolic phenotype [39]. Therefore, transparent reporting in arterial calcification studies is crucial for ensuring comparability of results. The finding that Sprague-Dawley rats exhibit higher arterial calcification than Wistar rats is a new discovery, as strain-specific calcification tendencies have not been reported previously. However, because the analyzed publications lack direct comparisons between strains, more research is needed to understand this finding.

The variation in arterial calcification effect sizes across model types likely reflects methodological differences. Because of the limited number of studies and missing data on key variables, conducting a reliable multiple meta-regression to account for this variability was not possible. Interestingly, some studies found similar arterial calcification outcomes despite major differences in factors believed to influence them, suggesting that adenine concentration, exposure duration, and nephrectomy period may not directly determine the severity of arterial calcification. The possibility of compensatory relationships between parameters warrants further investigation with larger datasets.

Publication bias analysis showed significant asymmetry in funnel plots and Egger’s test results, indicating selective reporting of positive findings. The presence of negative SMD mainly in intermediate control groups supports this conclusion. Additionally, large differences in experimental and control groups suggest that factors beyond calcification-induction protocols may affect study outcomes.

Although pooled analyses suggested greater arterial calcification in nephrectomy models than in adenine-based approaches, this difference was not consistently observed in direct comparisons. In fact, a previous study showed similar aortic calcium levels in adenine- and nephrectomy-treated Wistar rats fed a phosphate-rich diet [40]. At the same time, another study reported twice as much aortic calcium in rats with nephrectomy and a phosphate-enriched diet compared to the adenine model with a calcium/phosphate-enriched diet [41]. Notably, the adenine group and control group exhibited a similar level of calcification.

The generally lower variation coefficients in nephrectomy groups reported in the present study support their higher reliability for modeling CKD-dependent arterial calcification. Control groups across all model types showed significant variation, highlighting the natural differences in baseline tissue calcium measurements in rats. This emphasizes the importance of carefully selecting control groups and standardizing experimental design. The noticeable differences in control groups indicate that natural biological variability and environmental factors likely have a significant impact on experimental outcomes, regardless of the intervention being tested.

Several limitations must be acknowledged in our meta-analysis. First, the frequent omission of essential experimental parameters compromised thorough data collection and analysis, as shown in the risk of bias assessment. As a result, the meta-analysis includes only a subset of the studies conducted and shows significant methodological differences. The broad subgroup definitions, needed due to limited data, may mask important procedural variations. Second, although calcium content does not fully reflect the structural characteristics of ectopic calcifications and may include unrelated calcium phosphate precipitates, it was the most commonly used quantifiable measure of calcification, providing a way to harmonize studies. Third, the overrepresentation of certain laboratories and their methods could introduce bias. Finally, the localization of the calcification sites was evaluated as classified by the authors.

Improving understanding of CKD-MBD pathophysiology through experimental CKD models, including assessments of bone parameters in studies focused on arterial calcification, would be beneficial. Although some studies have commendably adopted this approach, we suggest that future research systematically include bone evaluations to gain a more complete view of CKD-MBD and to track potential skeletal effects of anti-calcifying treatments.

These findings highlight the important need for better data availability and standardized reporting guidelines. Calcium measurements should be fully reported, including sample size, standard deviation, detailed methodology, the vascular bed analyzed, and data for control groups. The timing and specific protocol of interventions should be clearly described, with all factors that might influence calcification status carefully documented. Ideally, diet information should include catalog numbers for complete nutrient and mineral traceability; for custom diets, the composition should be detailed. Based on a systematic analysis of the literature, we provide the following recommendations for establishing rat models of CKD-associated arterial calcification. These guidelines are designed to maximize effect size and minimize experimental variability, thereby supporting reproducible and translational research. Considering the calcification effect size and variation, subtotal nephrectomy in Sprague-Dawley rats combined with dietary phosphate (1.2%) reliably induces arterial calcification. It is important to note that surgical expertise is necessary. Current evidence does not support an additive effect of vitamin D supplementation in this model. However, differences in vitamin D sources, subforms, and dosages across studies limit definitive conclusions.

Adenine-based models provide a non-surgical alternative but are associated with greater variability, requiring thorough power analysis and larger sample sizes for reliable results. 0.3% adenine combined with 1.2% phosphate over eight weeks may effectively induce arterial calcification. Although male rats exhibited more pronounced arterial calcification, both sexes should be included in experimental designs in accordance with current animal research guidelines. This approach will enhance the translational relevance and address potential sex-specific mechanisms.

We suggest that studies include sufficient details to evaluate risk of bias, such as housing conditions, blinding, and basic animal characteristics (e.g., sex). Researchers are encouraged to adhere to established reporting guidelines and checklists, such as ARRIVE [42] or PREPARE [43], to promote transparency and reproducibility in animal research.

Improving data reporting will support high-quality meta-analyses and increase the translational value of preclinical arterial calcification research.

## Supplementary Information

Supplementary Material 1.

Supplementary Material 2.

## Data Availability

All extracted data are available in the supplemental tables.

## Abbreviations

Ad: Adenine
Ca: Calcium
CKD: Chronic kidney disease
CKD-MBD: Chronic kidney disease-mineral bone disorder
CVD: Cardiovascular diseases
FGF23: Fibroblast growth factor-23
P: Phosphate
PTH: Parathyroid hormone
SMD: Standardized mean difference
TNAP: Tissue-nonspecific alkaline phosphatase
VitD: Vitamin D
