# Supplementary material for: Rat models for arterial calcification associated with chronic kidney disease: a systematic review and meta-analysis
Source: BMC Cardiovasc Disord. 2026 Jan 27;26:82. doi: 10.1186/s12872-025-05471-4 (PMC12836979; doi:10.1186/s12872-025-05471-4)
Supplement: Supplementary file 2 — Supplementary Material 2. [file 12872_2025_5471_MOESM2_ESM.docx]

**Supplementary Figures**


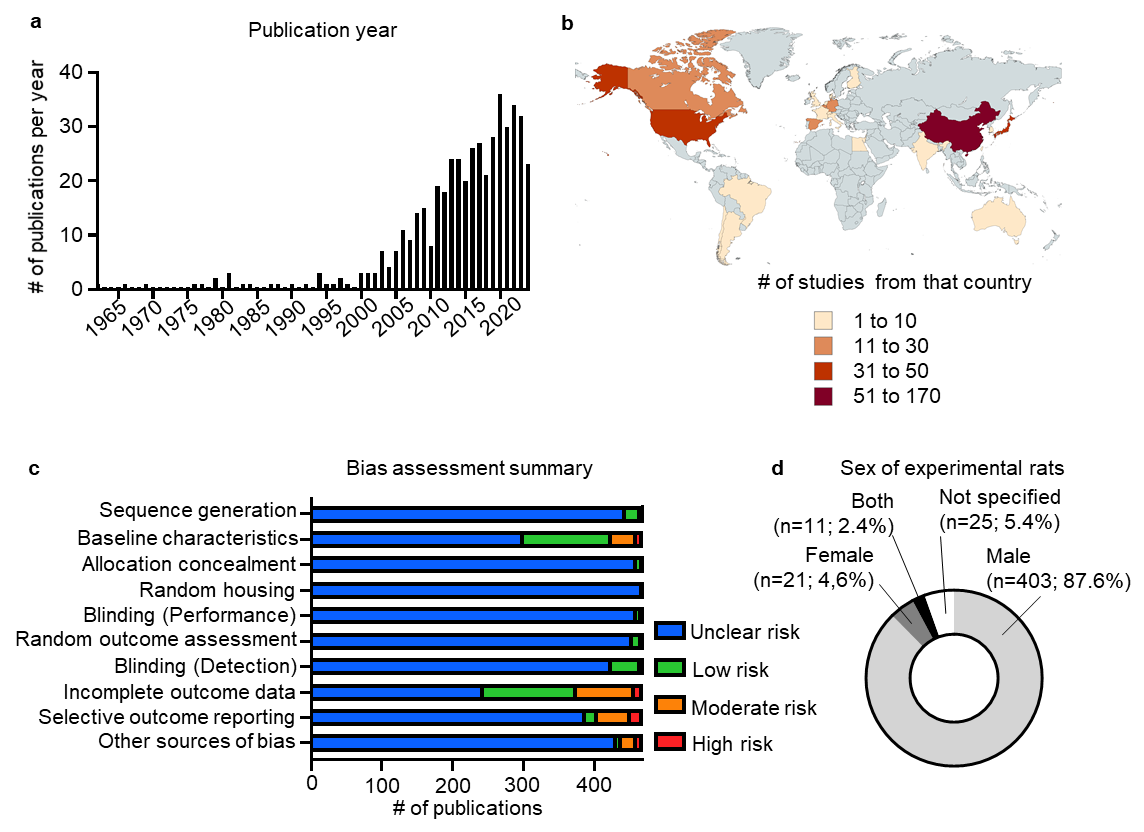


**Supplementary Figure 1. General data of the 470 publications included in the systematic review.** a) Distribution of year of publication (2024 included publications until 01.10.24). b) World map showing the origin of the animal experiments as far as identified (based on the institution approving the animal procedure). Created with mapcharts.net. c) Summary of risk of bias assessment. Numbers of publications are assigned a low (green), moderate (orange), high (red), or unclear (blue) risk of bias in the individual bias categories. d) Sex of the experimental animals after exclusion of studies focusing on sex-specific subjects. n indicates the number of publications.


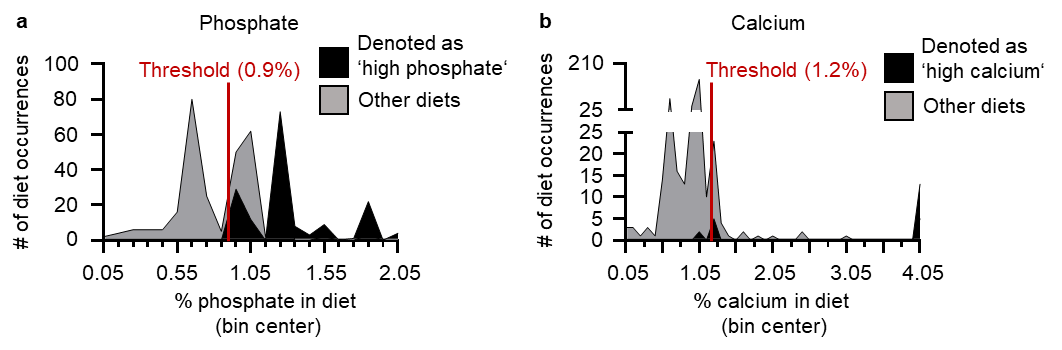


**Supplementary Figure 2. Phosphate and calcium percentage in the used diets.** a) The phosphate concentration of diets labeled as high phosphate diet (or similar terminology) (black) and all other diets (gray). b) The calcium concentration of diets labeled as high calcium diet (or similar terminology) (black) and all remaining diets. The y-axis shows the number of occurrences of each concentration across all diets. Percentages are rounded to one decimal place, so bins with a width of 0.1 were used. The bin centers (.05) are shown on the x-axis. Only diets with specified concentrations were included, while others were excluded. The red line marks the threshold for a high phosphate or high calcium diet, as defined for our analysis.


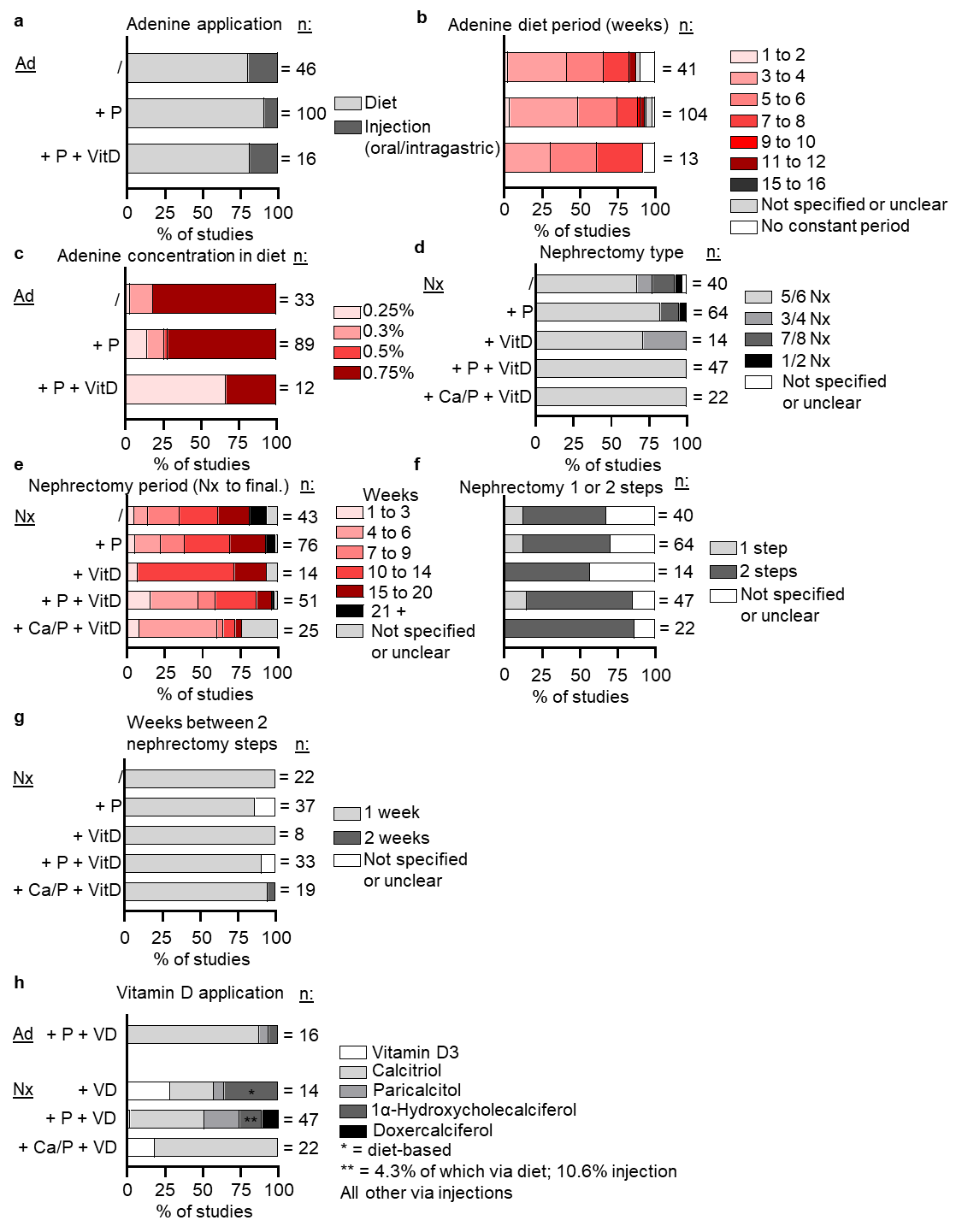


**Supplementary Figure 3. Additional general CKD-based arterial calcification model parameters.** a) Method of adenine administration. b) Period of adenine diet administration (if diet was used). ‘No constant period‘ if an alternating feeding pattern was applied. c) Adenine concentration in the adenine diet. d) Subtotal nephrectomy type. e) Period between finalization of subtotal nephrectomy and sacrifice in weeks. f) Subtotal nephrectomy performed in one or two steps. g) Time between subtotal nephrectomy steps in weeks (if two steps). h) Vitamin D derivative used for treatment and mode of application. n indicates the number of studies. Ad: Adenine; Nx: subtotal nephrectomy. /: no other treatment; P: high phosphate; VitD: Vitamin D (including several chemical derivatives); Ca: high calcium.


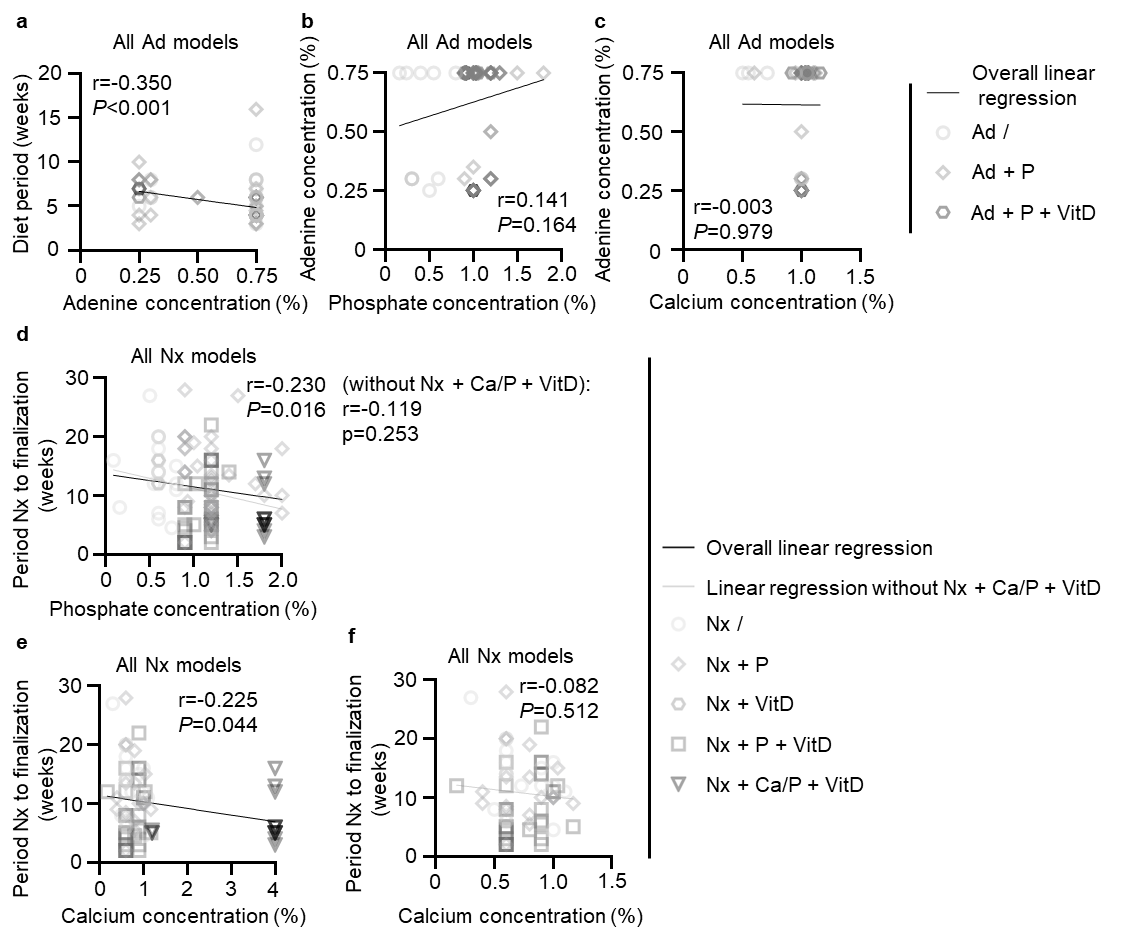


**Supplementary Figure 4. Associations between model parameters in combined adenine (Ad) and subtotal nephrectomy (Nx) models.** a) Between the period of the adenine diet and the adenine diet concentration. Only models using a constant adenine diet are assessed; no alternating feeding schemes of adenine injection. b-c) Association between adenine concentration and dietary phosphate or calcium. d) Period between Nx finalization versus diet phosphate concentration or calcium concentration (e). Correlation analysis was performed with all groups or excluding Nx + Ca/P + VitD. f) shows e) without Nx + Ca/P + VitD. Only studies with both parameters known are plotted. Data points are displayed with reduced opacity to reveal overlapping values. Several superposed data points with the same values, therefore, appear more saturated. A linear regression through the data points was calculated, and the correlation was tested statistically. Two-sided p-value is plotted. P = phosphate diet. Ca = Calcium diet. VitD = Vitamin D.


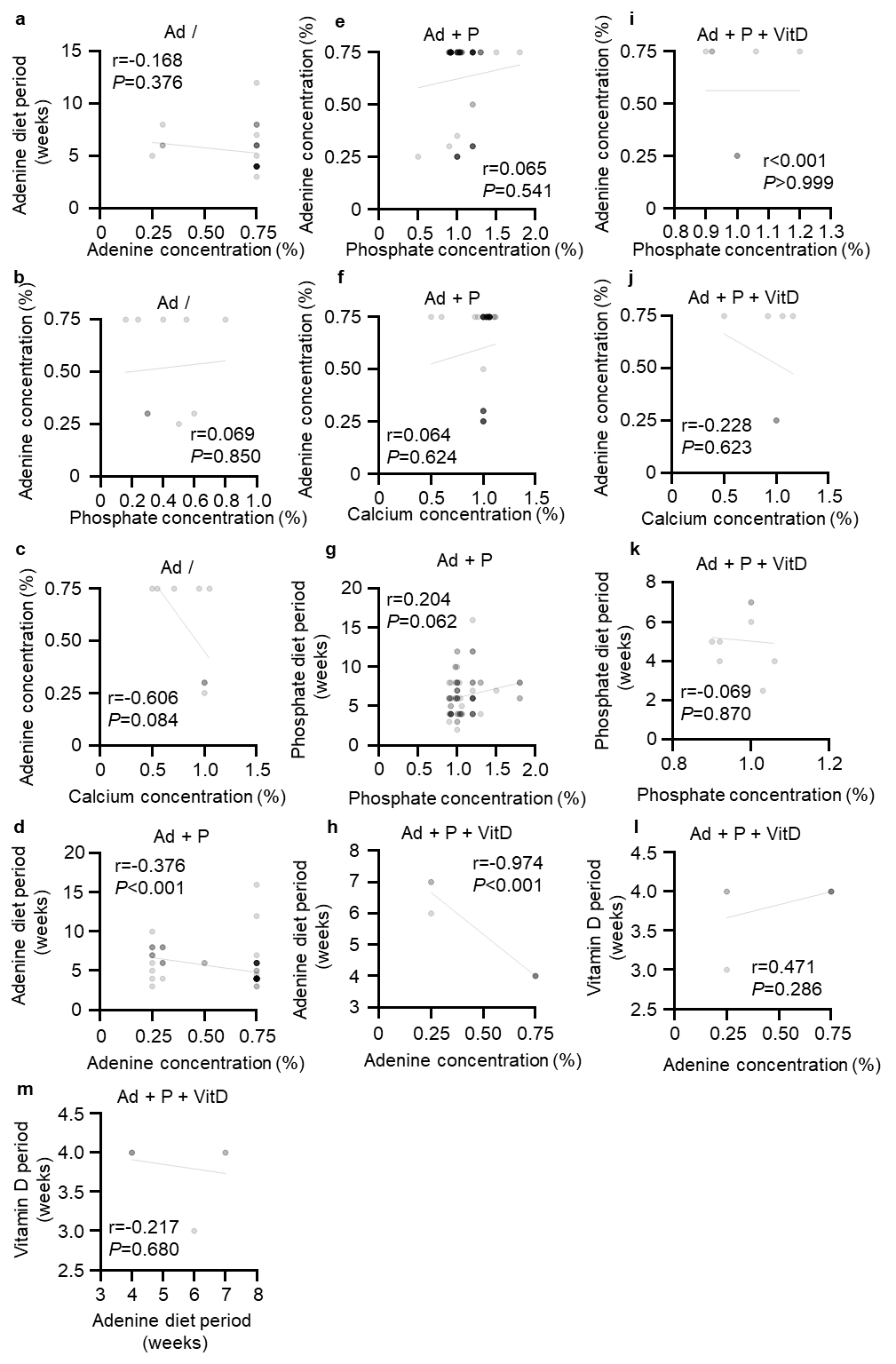


**Supplementary Figure 5. Associations between parameters in combined adenine** **(Ad) models.** Parameters as indicated on the axes. a-c) Ad without further treatment. d-g) Ad + phosphate diet. h-m) Ad + phosphate diet + Vitamin D. Only studies with both parameters known are plotted. Data points are displayed with reduced opacity to reveal overlapping values. Several superposed data points with the same values, therefore, appear more saturated. A linear regression through the data points was calculated, and the correlation was tested statistically. Two-sided p-value is plotted. P = phosphate diet. Ca = Calcium diet. VitD = Vitamin D.


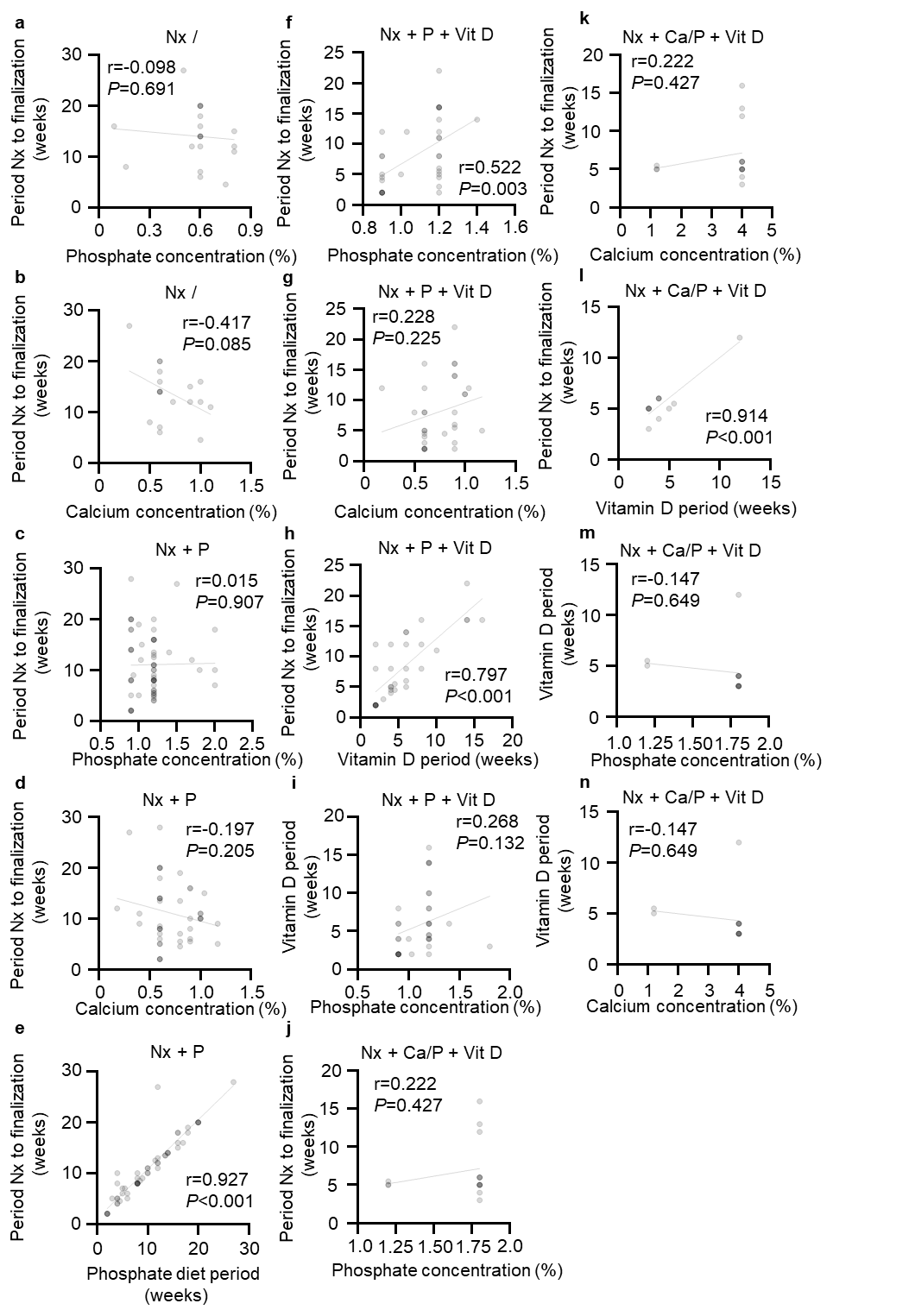


**Supplementary Figure 6. Associations between parameters in combined subtotal nephrectomy (Nx) models.** Parameters as indicated on the axes. a-b) Nx without further treatment. c-e) Nx + phosphate diet. f-i) Nx + phosphate diet + vitamin D. j-n) Nx + calcium/phosphate diet + vitamin D. Nx + vitamin D not shown because it comprises very few data points. Data points are displayed with reduced opacity to reveal overlapping values. Several superposed data points with the same values, therefore, appear more saturated. A linear regression through the data points was calculated, and the correlation was tested statistically. Two-sided p-value is plotted. P = phosphate diet. Ca = Calcium diet. Vit D = Vitamin D.


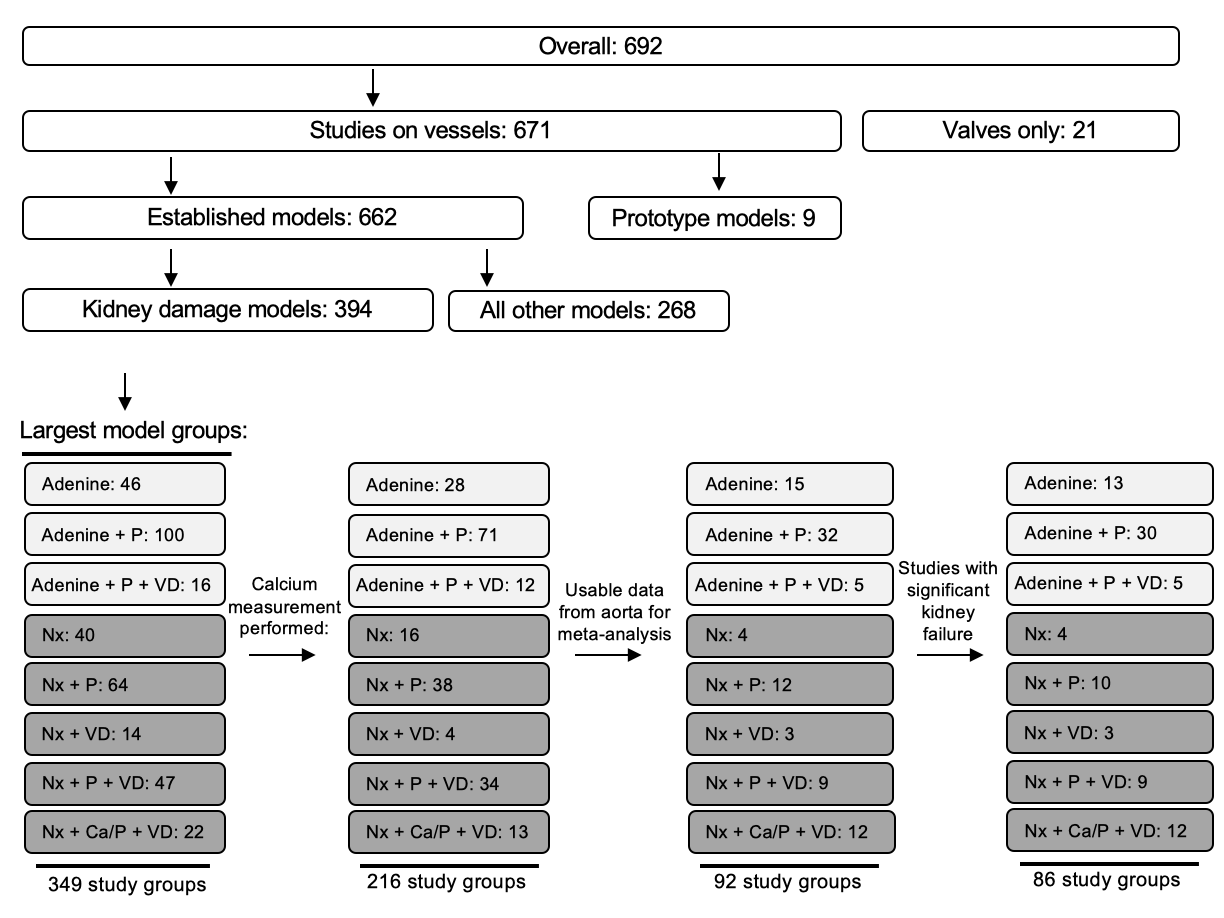


**Supplementary Figure 7. Scheme of study group selection for meta-analysis.** Overall, 692 study groups have been identified from the 470 publications included in the systematic review. 662 study groups investigated vessel calcification using established models. Most of these analyzed calcification as a comorbidity of renal destruction. The eight largest model groups, representing 349 study groups, were identified. For the meta-analysis, study groups that a) performed tissue calcium measurement, b) presented aortic tissue data in a statistically usable manner, and c) successfully induced renal obstruction (verified by blood creatinine, urea, BUN, or GFR measurements) were included. Ad: Adenine; Nx: subtotal nephrectomy. /: no other treatment; P: high phosphate; VD: Vitamin D (including several chemical derivatives); Ca: high calcium.


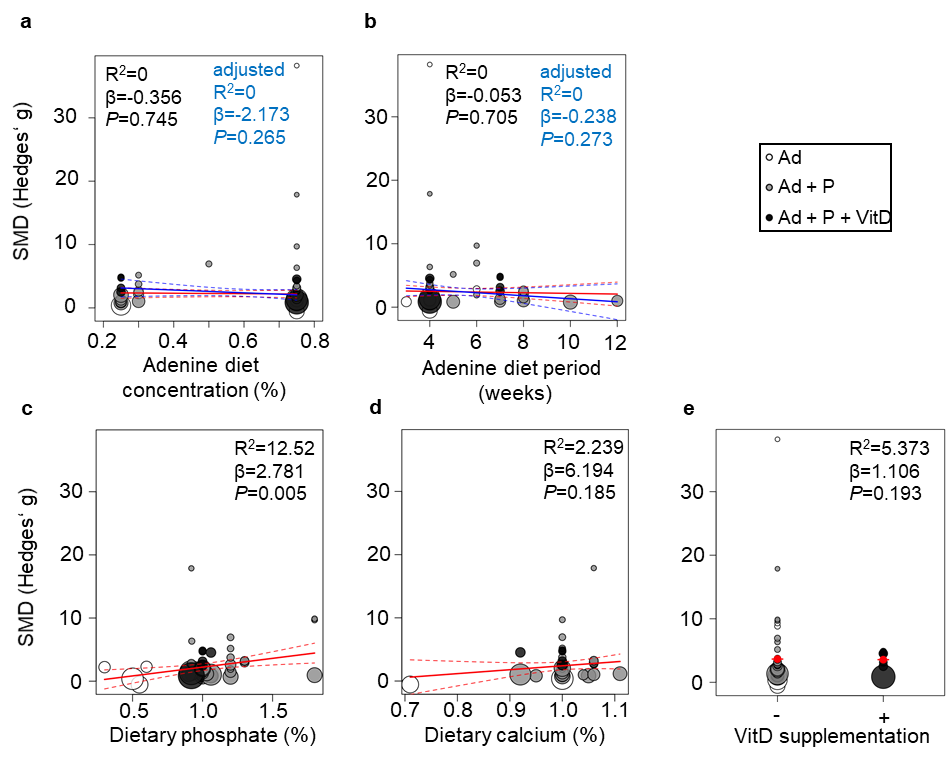


**Supplementary Figure 8. Meta-regression of adenine models.** Aortic calcium measurement data were calculated to standard mean difference (SMD (Hedges’g)). Model type subgroups were merged, and weighted meta-regressions were calculated to test the influence of a moderator variable (adenine concentration in diet (a), adenine diet period (b), phosphate or calcium concentration in the diet (c-d); vitamin D supplementation (e); as far as specified) on the SMD. A simple linear regression was performed to predict the effect size (red line) as a function of one variable. Dashed red lines indicate the 95% confidence interval. (a) Adenine concentration in the diet was adjusted for the adenine diet period, and (b) the adenine diet period was adjusted for adenine concentration using a multiple regression model (blue lines and text). n=43 data points of diet-based, constant adenine regimens with both dietary concentration and period being specified were used for the multiple regression model. Simple linear regressions utilized all clearly specified data points for the respective variable. Due to the high heterogeneity of vitamin D supplementation (exact compound, dose, frequency, and method of administration), general supplementation was treated as a binary variable for simplification. Dot size corresponds to statistical weight. Ad: Adenine; /: no other treatment; P: high phosphate; VitD: Vitamin D (including several chemical derivatives).


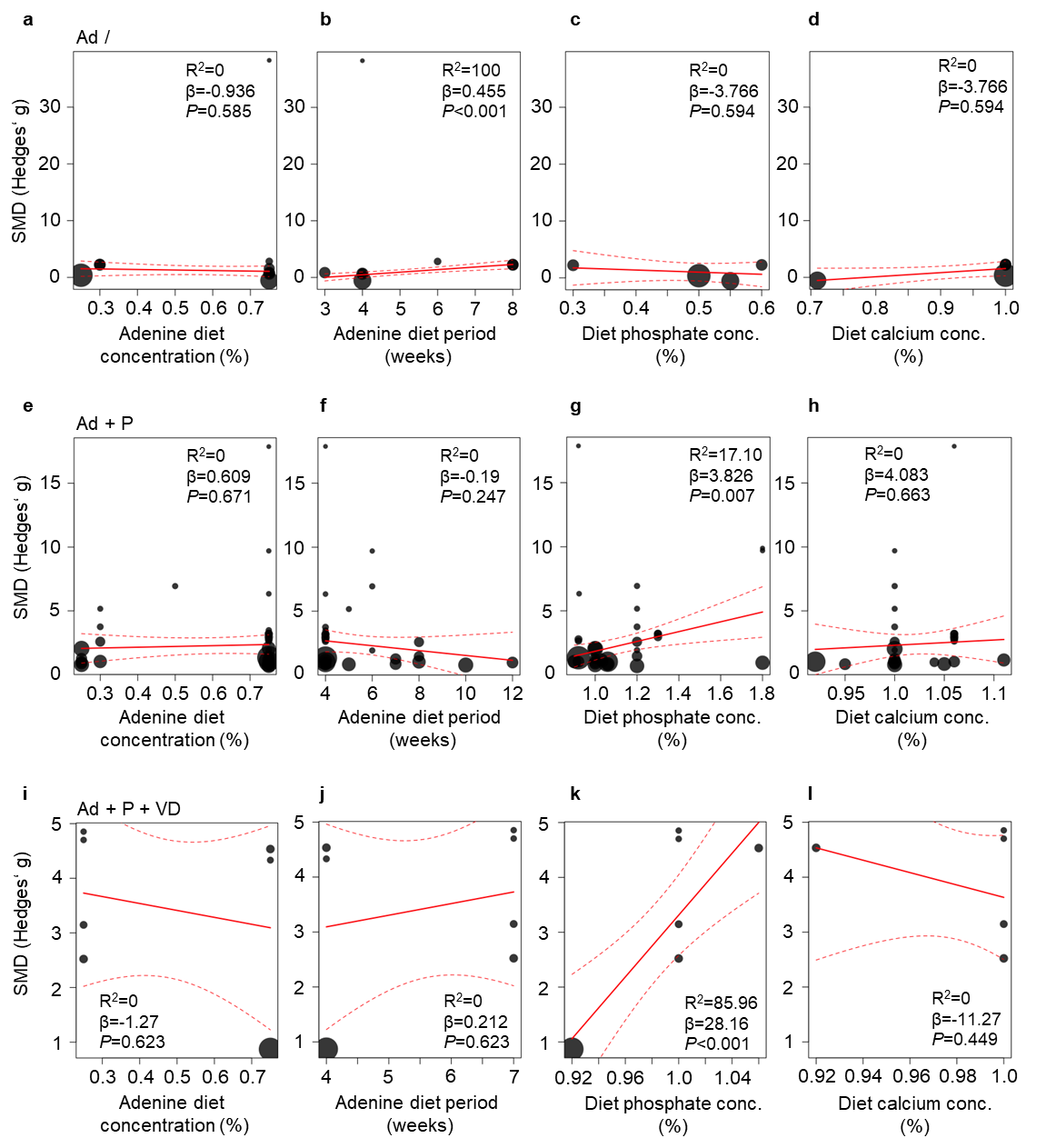


**Supplementary Figure 9. Meta-regression to test the influence of a moderator variable** (adenine concentration in diet, adenine diet period, dietary phosphate or calcium concentration as far as specified) on the standard mean difference (SMD (Hedges’g)). Each model group was analyzed separately. a-d) Adenine, e-h) Adenine + high phosphate, i-l) Adenine + high phosphate + Vitamin D (including several chemical derivatives). Red line shows the predicted effect size. Dashed red lines indicate the 95% CI. Dot size corresponds to statistical weight.


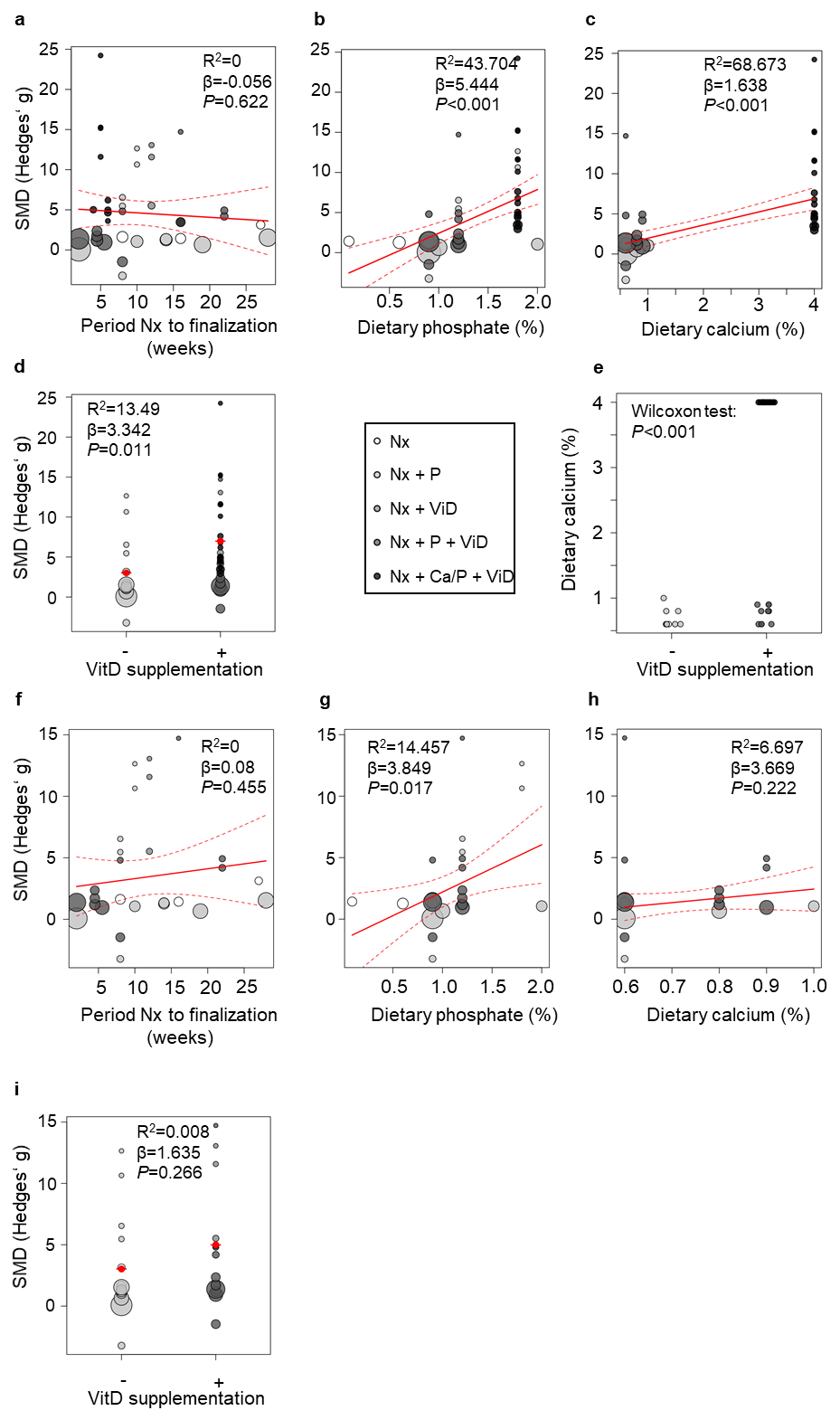


**Supplementary Figure 10: Meta-regression of nephrectomy models.** Aortic calcium measurement data were calculated to standard mean difference (SMD (Hedges’g)). Model type subgroups were merged and weighted meta-regressions were calculated to test the influence of a moderator variable (period from nephrectomy to finalization (a), phosphate or calcium concentration in the diet (b-c); vitamin D supplementation (d); as far as specified) on the SMD. A simple linear regression was performed to predict the effect size (red line) as a function of one variable. Dashed red lines indicate the 95% confidence interval. Simple linear regressions utilized all clearly specified data points for the respective variable. Due to the high heterogeneity of vitamin D supplementation (exact compound, dose, frequency, and method of administration), general supplementation was treated as a binary variable for simplification. As the studies with vitamin D include the highest calcium concentrations (e), the analyses were repeated after exclusion of the group Nx + Ca/P + VitD (f-i). Dot size corresponds to statistical weight. Nx: subtotal nephrectomy. /: no other treatment; P: high phosphate; VitD: Vitamin D (including several chemical derivatives); Ca: high calcium.


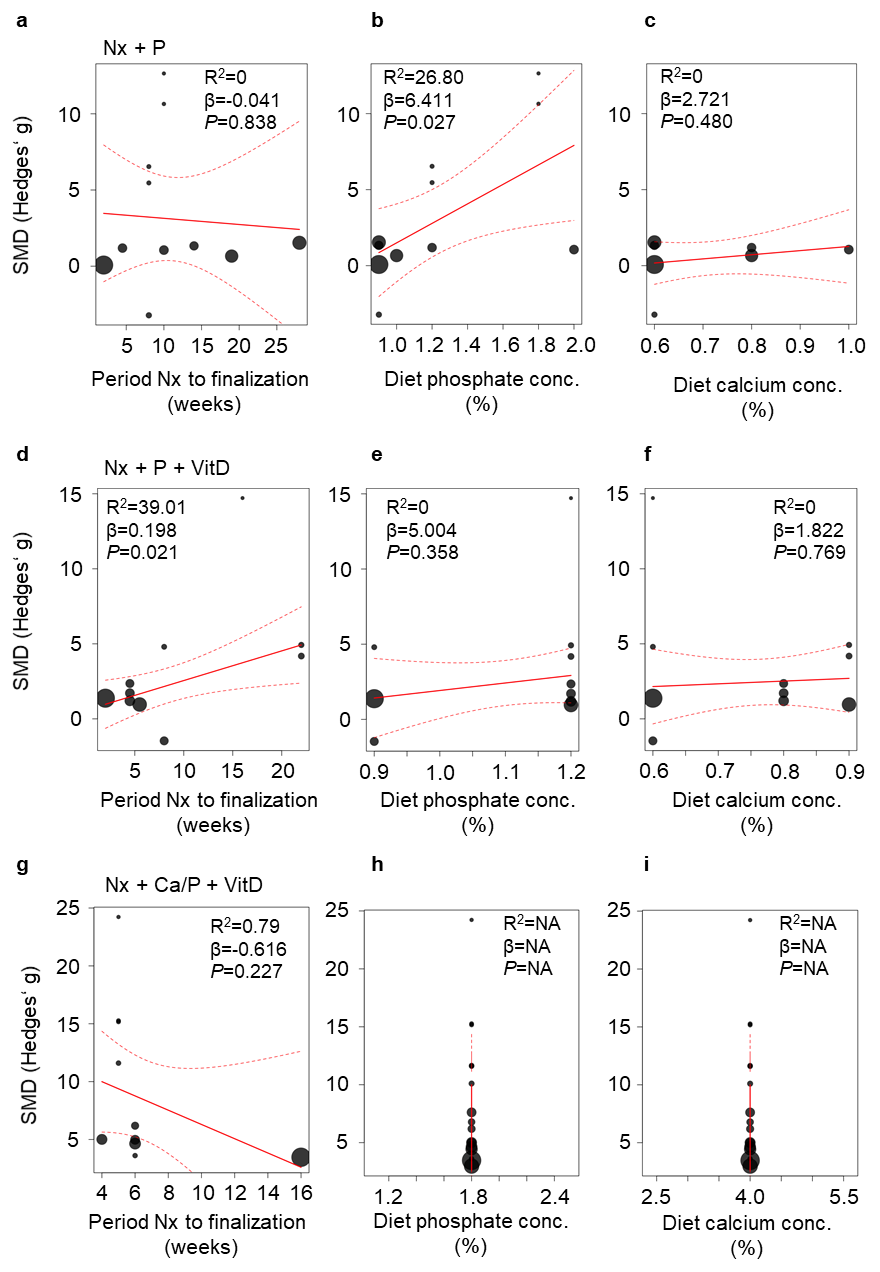


**Supplementary Figure 11. Meta-regression to test the influence of a moderator variable** (period between Nx finalization and sacrifice, dietary phosphate or calcium concentration as far as specified) on the standard mean difference (SMD). Each model group analyzed separately. a-c) Subtotal nephrectomy + phosphate diet, d-f) Subtotal nephrectomy + phosphate diet + Vitamin D (including several chemical derivatives), g-i) Subtotal nephrectomy + calcium/phosphate diet + Vitamin D (including chemical derivatives). Subtotal nephrectomy only and subtotal nephrectomy + Vitamin D are omitted because they only contained 2-4 data points. Red line shows the predicted effect size. Dashed red lines indicate the 95% CI. Dot size corresponds to statistical weight. Nx: subtotal nephrectomy; P: high phosphate; VitD: Vitamin D (including several chemical derivatives); Ca: high calcium.


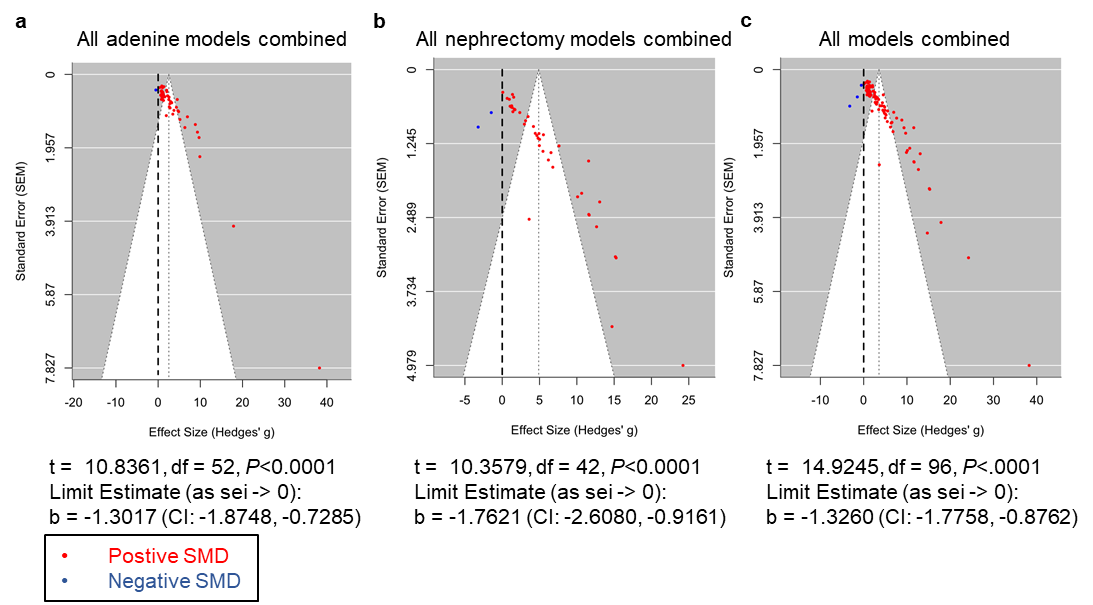


**Supplementary Figure 12. Funnel plots and regression test** (Egger’s test) for asymmetry. For all adenine (Ad) models (a), all subtotal nephrectomy (Nx) models (b), and all models combined (c). Model: weighted regression with multiplicative dispersion. All data sets used in the meta-analysis are represented in this statistic. Thick dashed line: Effect size (standardized mean difference (SMD); Hedges’ g) = 0. Thin dashed line = reference line based on the pooled effect calculated with a random effects model. df = degrees of freedom.


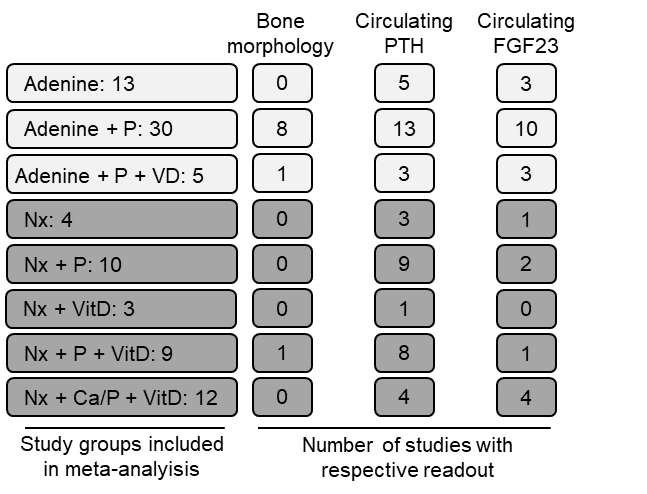


**Supplementary Figure 13.** Quantitative assessment of the studies reporting data on non-vascular/bone-associated metrics of CKD-MBD (bone morphology, PTH, FGF23). Nx: subtotal nephrectomy; P: high phosphate; VitD: Vitamin D (including several chemical derivatives); Ca: high calcium.
